# Intravital Imaging to Monitor Therapeutic Response in Moving Hypoxic Regions Resistant to PI3K Pathway Targeting in Pancreatic Cancer

**DOI:** 10.1016/j.celrep.2018.05.038

**Published:** 2018-06-13

**Authors:** James R.W. Conway, Sean C. Warren, David Herrmann, Kendelle J. Murphy, Aurélie S. Cazet, Claire Vennin, Robert F. Shearer, Monica J. Killen, Astrid Magenau, Pauline Mélénec, Mark Pinese, Max Nobis, Anaiis Zaratzian, Alice Boulghourjian, Andrew M. Da Silva, Gonzalo del Monte-Nieto, Arne S.A. Adam, Richard P. Harvey, Jody J. Haigh, Yingxiao Wang, David R. Croucher, Owen J. Sansom, Marina Pajic, C. Elizabeth Caldon, Jennifer P. Morton, Paul Timpson

**Affiliations:** 1 Garvan Institute of Medical Research and The Kinghorn Cancer Centre, Cancer Division, Sydney, NSW 2010, Australia; 2 St Vincent’s Clinical School, Faculty of Medicine, University of NSW, Sydney, NSW 2010, Australia; 3 Developmental and Stem Cell Biology Division, Victor Chang Cardiac Research Institute, Sydney, NSW 2010, Australia; 4 School of Biotechnology and Biomolecular Science, University of New South Wales, Sydney, NSW 2033, Australia; 5 Australian Centre for Blood Diseases, Monash University, Melbourne, VIC 3004, Australia; 6 Department of Bioengineering, University of Illinois, Urbana-Champaign, Urbana, IL 61801, USA; 7 School of Medicine and Medical Science, University College Dublin, Belfield, Dublin 4, Ireland; 8 Cancer Research UK Beatson Institute, Switchback Road, Bearsden, Glasgow G61 1BD, UK; 9 Institute of Cancer Sciences, University of Glasgow, Glasgow G61 1QH, UK

**Keywords:** pancreatic cancer, intravital imaging, hypoxia, FRET, pro-drug, PI3K pathway, nanoparticles, PLIM, Akt, AZD2014

## Abstract

Application of advanced intravital imaging facilitates dynamic monitoring of pathway activity upon therapeutic inhibition. Here, we assess resistance to therapeutic inhibition of the PI3K pathway within the hypoxic microenvironment of pancreatic ductal adenocarcinoma (PDAC) and identify a phenomenon whereby pronounced hypoxia-induced resistance is observed for three clinically relevant inhibitors. To address this clinical problem, we have mapped tumor hypoxia by both immunofluorescence and phosphorescence lifetime imaging of oxygen-sensitive nanoparticles and demonstrate that these hypoxic regions move transiently around the tumor. To overlay this microenvironmental information with drug response, we applied a FRET biosensor for Akt activity, which is a key effector of the PI3K pathway. Performing dual intravital imaging of drug response in different tumor compartments, we demonstrate an improved drug response to a combination therapy using the dual mTORC1/2 inhibitor AZD2014 with the hypoxia-activated pro-drug TH-302.

## Highlights

• Hypoxia presents a moving pocket of resistance in pancreatic ductal adenocarcinoma.
• Dual intravital imaging allows live tracking of drug response in hypoxic regions.
• Combination with a hypoxia-activated pro-drug alleviates resistance.
• This has implications for combatting resistance in a broad range of therapies.

## Graphical Abstract

**Figure:**
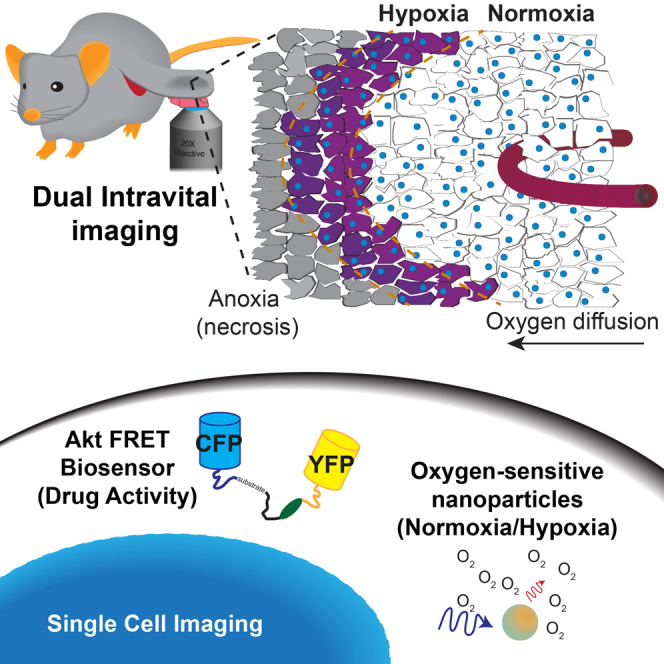

## Teaser

Intravital imaging facilitates the real-time tracking and targeting of moving hypoxic regions within pancreatic ductal adenocarcinoma. Using this approach, Conway et al. alleviate hypoxia-induced resistance to a dual mTORC1/2 inhibitor AZD2014, improving PI3K pathway inhibition and demonstrating a powerful dual imaging modality applicable to targeting other pathways and cancers.

## Introduction

A recurring feature in malignant cancers is the aggressive alterations that cells undergo in order to survive in a low-oxygen (hypoxic) environment. This hypoxic environment is commonly a result of insufficient tumor vascularity and is strongly associated with increased radioresistance, chemoresistance, and metastasis (Rofstad et al., 2007, Sullivan et al., 2008, Wilson and Hay, 2011). Among those cancers with a propensity for high levels of tumor hypoxia, pancreatic ductal adenocarcinoma (PDAC) has a well-established hypoxic signature, which is predictive of poorer patient prognosis (Chang et al., 2011, Miller et al., 2015). PDAC has a poor 5-year survival of less than 8% and is predicted to be the second leading cause of cancer-related deaths by 2030 (Rahib et al., 2014, Siegel et al., 2017). One approach to improve patient outcome has been to target hypoxic tumor regions in order to improve both drug penetrance and response (Chen et al., 2016, Sun et al., 2015). Hypoxia-activated pro-drugs (HAPs) provide an efficient method to specifically deliver cytotoxic agents to hypoxic tumor regions, diminishing the off-target effects from conventional small-molecule therapeutics (O’Connor et al., 2016, Wilson and Hay, 2011). These HAPs may also affect normoxic tumor regions by virtue of a local bystander effect and in this way provide an even more versatile approach for targeted delivery (Abbattista et al., 2015, Sun et al., 2015). Cytotoxic mustard-based HAPs, such as TH-302 (Meng et al., 2012, Sun et al., 2015) and PR-104 (Abbattista et al., 2015), are currently the most widely applied, building on the ushering work of tirapazamine and related derivatives (Kovacs et al., 1999, Wilson and Hay, 2011). Recently, we have seen an emergence of bioreductive groups attached to targeted therapeutics, concomitant with improvements in their synthesis and sensitivity (O’Connor et al., 2016, Wilson and Hay, 2011). However, HAPs are generally insufficient for use as monotherapies, and current clinical trials focus on their application in combination with chemotherapeutics, radiotherapy, or targeted therapies (Borad et al., 2015, Van Cutsem et al., 2016, Wilson and Hay, 2011).

In recent work, we demonstrated that targeting of the phosphatidylinositol 3-kinase (PI3K) pathway with the dual mTORC1/2 inhibitor AZD2014 presents a therapeutic opportunity for PDAC, equivalent to the standard-of-care gemcitabine (Driscoll et al., 2016). This built upon our previous work, whereby the mTORC1 inhibitor rapamycin showed improved efficacy in tumors with high PI3K pathway activity (Kennedy et al., 2011, Morran et al., 2014). There is a well-established role for the PI3K pathway in hypoxia, which is linked with chemoresistance, metabolic reprogramming, and angiogenesis (Chae et al., 2016, Guo et al., 2016). Here, we identify a phenomenon whereby two aggressive PDAC models were resistant to three clinically relevant PI3K pathway inhibitors, when grown in a hypoxic environment. We investigate this resistance within a three-dimensional (3D) organotypic co-culture system (Conway et al., 2014, Morton et al., 2010, Vennin et al., 2017) and confirm that resistance to PI3K pathway inhibition only occurred in hypoxic cells invading into the matrix. Furthermore, we reveal that hypoxia moves transiently around the tumor *in vivo* and this pocket of resistance presents a potential moving target in PDAC.

To combat this hypoxia-induced resistance, we developed a combination therapy that combines the dual PI3K pathway inhibitor AZD2014 with the HAP TH-302. This combination reduces the hypoxia-induced resistance to AZD2014 *in vitro* and significantly inhibits tumor growth *in vivo*. To elucidate the improved outcomes from this combination therapy, we performed live intravital microscopy (IVM) of drug-target activity using an intramolecular Förster resonance energy transfer (FRET) biosensor for Akt activity (Komatsu et al., 2011). Monitoring drug response in individual cells by fluorescence lifetime imaging microscopy (FLIM)-FRET, in parallel with phosphorescence lifetime imaging microscopy (PLIM) of oxygen-sensitive nanoparticles (Conway et al., 2017, Kondrashina et al., 2012), enabled the stratification of single-cell responses within different tumor compartments. This demonstrated that regions with poorer drug response also had lower oxygen content. Furthermore, when tumor-bearing mice were treated in combination with the HAP TH-302 and AZD2014, Akt activity was further reduced, compared to AZD2014 monotherapy. Critically, these findings highlight both the value of combining targeted therapeutics with HAPs and the preclinical power of IVM in the drug discovery pipeline (Conway et al., 2014, Miller and Weissleder, 2017).

## Results

### Hypoxia Induces Resistance to PI3K Pathway Inhibition in PDAC

**Figure 1:**
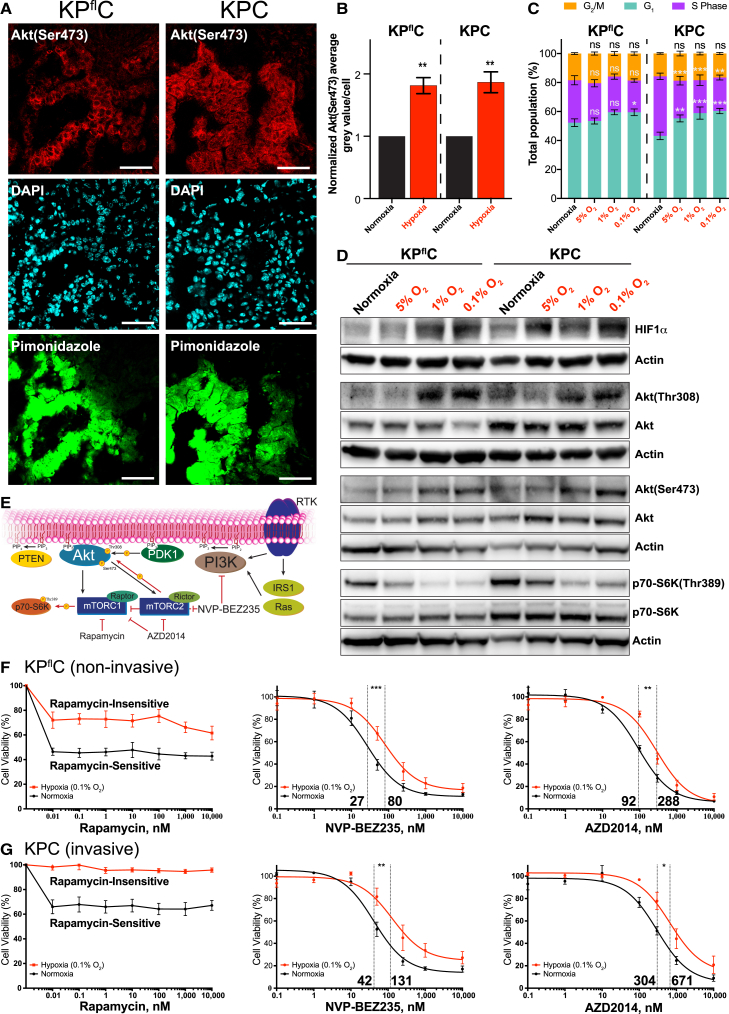
The Presence of Hypoxia and the Associated Molecular, Phenotypic, and Resistance Effects in the KP^fl^C and KPC Mouse Models of PDAC (A) Immunofluorescence staining of the GEM KP^fl^C and KPC PDAC mouse models for Akt(Ser473) (red), DAPI (cyan), and pimonidazole (green). Scale bars, 50 μm. (B) Quantification of Akt(Ser473) grey value/cell in pimonidazole negative (normoxic) and positive (hypoxic) regions (n = 5 tumors/mouse model). Mean ± SEM. p values are from a one-sample t test. (C) Propidium iodide staining of cell cycle phase distribution in the KP^fl^C and KPC primary PDAC cell lines. Mean ± SEM. p values were calculated using a two-way ANOVA with a Tukey correction for multiple comparisons. (D) Representative western blots of the KP^fl^C and KPC primary PDAC cell lines, incubated for 48 hr in normoxia or hypoxia (5%, 1%, or 0.1% oxygen; n = 5). (E) A simplified schematic of the PI3K pathway, indicating the targets of the PI3K pathway inhibitors used in this study. (F and G) IC_50_ curves demonstrating the response of KP^fl^C (F) and KPC (G) primary PDAC cell lines to the PI3K pathway inhibitors rapamycin, NVP-BEZ235, and AZD2014, in both normoxic (black lines) and hypoxic (0.1% oxygen, red lines) conditions (n = 3). An extra sum-of-squares F test was performed between the best-fit parameters of each curve. ^∗^p < 0.05, ^∗∗^p < 0.01, and ^∗∗∗^p < 0.001. See also Figure S1.

Hypoxia is a common feature of PDAC and are often associated with malignant disease (Chang et al., 2011, Miller et al., 2015). Hypoxia is regularly the result of a disorganized vascular network within the tumor and, in the extreme case of anoxia (no oxygen), may lead to necrosis (Wilson and Hay, 2011). To investigate the role of hypoxia in PDAC, we used the well-established non-invasive *LSL-Kras*^*G12D/+*^*, LSL-Trp53*^*fl/+*^*, Pdx1-Cre* (KP^fl^C) and invasive *LSL-Kras*^*G12D/+*^*, LSL-Trp53*^*R172H/+*^*, Pdx1-Cre* (KPC) genetically engineered mouse (GEM) models (Hingorani et al., 2003, Hingorani et al., 2005, Morton et al., 2010). These PDAC models possess driver mutations in *Kras* and loss or gain-of-function mutation of the tumor suppressor gene *Trp53*, which occur in ∼95% and ∼75% of human PDAC cases, respectively (Hingorani et al., 2005, Morton et al., 2010, Waddell et al., 2015). This altered p53 function has been associated with improved survival of cancer cells in hypoxic conditions (Leszczynska et al., 2015). However, we found no significant difference in the fraction of Ki67-positive cells between hypoxic and normoxic regions of tumors in either model, as stratified by pimonidazole staining (Figure S1A; quantified in Figure S1B) (Varia et al., 1998). Importantly, we observed a significant upregulation in Akt activity in hypoxic regions of the GEM PDAC models (Figure 1A; quantified in Figure 1B), in line with previous reports (Chae et al., 2016, Guo et al., 2016).

Primary cell lines with loss or gain of function of p53 (Figure S1C), previously isolated from both PDAC mouse models, were assessed for their response to hypoxia at 5%, 1%, and 0.1% oxygen (Morton et al., 2010). A small decrease in S phase at decreasing oxygen levels was observed for both cell lines when performing cell cycle analysis by fluorescence-activated cell sorting (FACS) (Figure 1C). To assess whether the slight reduction in S phase conferred a decrease in cell number, we quantified cells in 0.1% oxygen and found no significant decrease in cell number, compared to normoxia (Figure S1D). Furthermore, decreasing oxygen levels were not found to have a significant effect on apoptosis in either of the PDAC cell lines, assessed by Annexin V staining (Figure S1E).

As anticipated, we observed a significant increase in HIF1α stabilization at 5%, 1%, and 0.1% oxygen (Figure 1D; quantified in Figure S1F), and a graded increase in pimonidazole adduct formation (Figure S1G; quantified in Figure S1H). Having established a hypoxic response, we assessed the activity of the PI3K pathway and identified a significant upregulation of Akt activity in hypoxia in both cell lines (Figure 1D; quantified in Figures S1I and S1J). Conversely, looking downstream of mTORC1, phosphorylation of p70-S6K was significantly reduced, consistent with previous reports (Figure 1D; quantified in Figure S1K) (Faes et al., 2016). These data confirm a role for the PI3K pathway in the hypoxic response within our models and prompted assessment of therapeutic inhibition of the PI3K pathway within this hypoxic environment.

We employed three clinically relevant inhibitors of the PI3K pathway, namely, rapamycin, NVP-BEZ235, and AZD2014 (Figure 1E). These inhibitors allowed us to target multiple levels of the PI3K pathway to provide a broader assessment of therapeutic response (Figures 1E–1G). Evaluating the half-maximal inhibitory concentration (IC_50_) for each inhibitor in both hypoxia and normoxia, we found that primary PDAC cell lines from each model showed hypoxia-induced resistance to all three PI3K pathway-targeted therapeutics (Figures 1F and 1G).

### 3D Assessment of Hypoxia-Induced Resistance in Invasive KPC PDAC Cells

**Figure 2:**
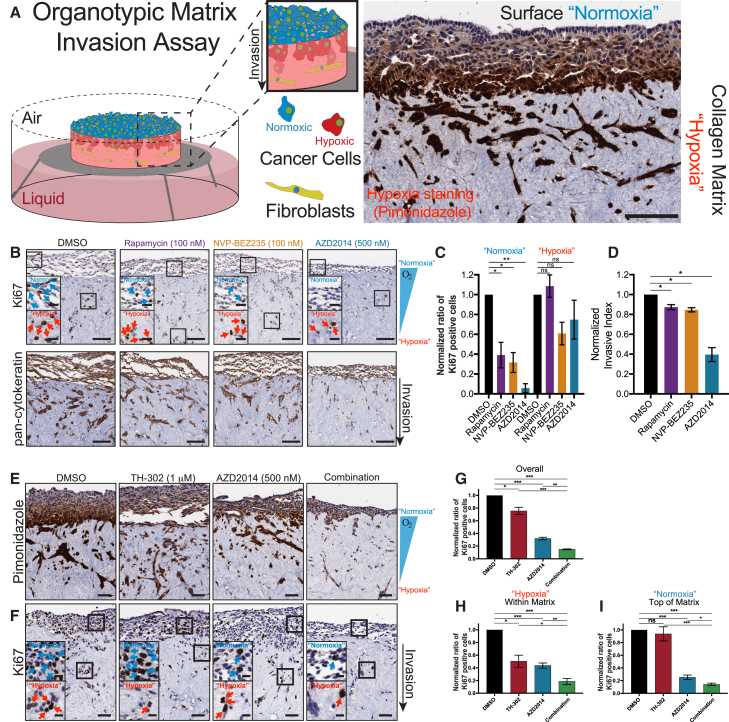
Organotypic Invasion Assay of KPC Cells Treated with PI3K Pathway Inhibitors and/or TH-302 (A) A schematic representation of the organotypic matrix assay and a representative area of invading KPC cells stained with pimonidazole (a marker of hypoxia). (B) Representative images of invading KPC cells stained with the epithelial cell marker pan-cytokeratin or the proliferative marker Ki67. Scale bars, 100 μm; insets, 25 μm. (C) Normalized Ki67 staining from the same experiments scored for cells on top of the organotypic matrix (normoxia) and cells invading into the matrix (hypoxia). Mean ± SEM. (D) Normalized invasive index of KPC cells treated with NVP-BEZ235, AZD2014, or rapamycin (n = 3). Mean ± SEM. (E and F) Representative images of invading KPC cells stained with the hypoxia marker pimonidazole (E) or the proliferative marker Ki67 (F). Scale bars, 50 μm; insets, 10 μm. Mean ± SEM. (G) Quantification of Ki67-stained KPC cells invading into organotypic matrices (n=4). (H and I) Assessment of Ki67-stained KPC cells either on top of (H, normoxia) or invading into (I, hypoxia) organotypic matrices (n = 4). A one-sample t test was performed on normalized data in all panels. ^∗^p < 0.05, ^∗∗^p < 0.01, and ^∗∗∗^p < 0.001. See also Figure S2.

The KPC PDAC model recapitulates the invasive and metastatic phenotype that is frequently observed in the clinical management of this disease (Hingorani et al., 2003, Hingorani et al., 2005, Morton et al., 2010). We therefore used 3D organotypic invasion assays to assess the effects of targeting the PI3K pathway in the invasive KPC cells (Conway et al., 2014, Morton et al., 2010, Vennin et al., 2017). To confirm the presence of hypoxia within our 3D organotypic matrices, KPC cells were seeded and allowed to grow on fibroblast-contracted matrices for 4 days before mounting the matrices on an air-liquid interface, which promotes invasion toward the chemo-attractive media (Figure 2A) (Conway et al., 2014, Morton et al., 2010, Vennin et al., 2017). Interestingly, staining for hypoxia within the organotypic matrices demonstrated a clear gradient of decreasing oxygen content from the surface of the matrix (see “Normoxia,” Figure 2A) to the invading cells within the matrix (see “Hypoxia,” Figure 2A). To confirm that this gradient was not a result of poor diffusion of pimonidazole into the matrix, we treated organotypic matrices with the fluorescent probe laurdan, as well as the autofluorescent drug doxorubicin (Figures S2A and S2B). In both cases, fluorescence of each compound was visible in cells on the surface and within the matrix (Figures S2A and S2B). To further corroborate the presence of a hypoxic gradient, we stained our organotypics for expression of GLUT1 and lactate dehydrogenase (Figures S2C and S2D). These proteins are typically upregulated under hypoxic conditions and were found to be upregulated in hypoxia (5%, 1%, and 0.1% oxygen) in the KPC cells (Figure S2E; quantified in Figure S2F) (Shukla et al., 2017).

Next, cells invading into organotypic matrices were treated with each PI3K pathway inhibitor (Figure 2B). Supporting our IC_50_ curves, the cells on the surface of the matrix (“Normoxia”) demonstrated a significant reduction in Ki67 staining for all inhibitors, while cells invading into the organotypic matrices (“Hypoxia”) were resistant to the inhibitors (Figure 2B, inset: see blue arrows and note reduction in Ki67-positive cells in normoxia for all inhibitors, which was less evident in hypoxia [see red arrows]; quantified in Figure 2C). Furthermore, treatment with rapamycin and NVP-BEZ235 yielded a subtle yet significant decrease in invasion, while AZD2014 induced a robust anti-invasive effect (Figure 2B; quantified in Figure 2D). Taken together, we found a common hypoxia-induced resistance to PI3K pathway targeting within our 3D organotypic matrices.

### Therapeutic Response of PDAC Cells Treated with AZD2014 Is Improved by the HAP TH-302

After confirming the hypoxia-induced resistance to PI3K pathway inhibition within the 3D organotypic assay, we chose to combat this resistance in a combination therapy. An attractive option for targeting the resistant hypoxic compartment is through the use of HAPs, which provide additional benefits with minimal toxicity to normoxic tissues (Abbattista et al., 2015, Meng et al., 2012, Wilson and Hay, 2011). We selected the dual mTORC1/2 inhibitor AZD2014 to develop a combination therapy with the HAP TH-302. TH-302 has already been shown to improve mTOR-targeted therapies in renal cell carcinoma (Sun et al., 2015), and within our models, AZD2014 alone has demonstrated a clear survival benefit, equivalent to that of gemcitabine (Driscoll et al., 2016). Here, we identified an additional anti-invasive effect provided by AZD2014 (Figure 2D, see blue bar), which may address the clinical need to reduce invasion and metastasis in PDAC patients. We next assessed the effect of TH-302 alone on KPC cells (Figure S2G). As expected, TH-302 showed a greatly reduced IC_50_ in hypoxia, compared to normoxia, consistent with an increased potency in hypoxia (Figure S2G) (Meng et al., 2012). We then assessed the effect of TH-302 on the hypoxia-induced resistance of AZD2014 (Figures 2E–2I). Having established the presence of a hypoxic gradient within our organotypic matrices (Figure 2A), we assessed the effect of combining TH-302 with AZD2014 in a 3D environment (Figures 2E and 2F). In combination, the anti-proliferative effects of both inhibitors were significantly improved overall (Figure 2G, compare red and blue bars to green). Upon further analysis, we found hypoxia-induced resistance to AZD2014 for cells within the matrix (Hypoxia), which was significantly reduced in combination with TH-302 (Figure 2F, inset: see red arrows). In contrast, TH-302 treatment led to a 50% decrease in Ki67-positive cells within the matrix (Hypoxia), which did not occur on the surface of the matrix (Figure 2F, inset, see red/blue arrows; Figures 2H and 2I, compare black bars to red). Furthermore, we assessed the effect of AZD2014 on the PI3K pathway by staining matrices for the downstream stress response gene NDRG1(Thr346). This staining highlighted a significant downregulation of NDRG1 activity in the combination therapy, compared to AZD2014 alone (Figure S2H). Consistent with a role in stress response, NDRG1 was also shown to be upregulated in decreasing oxygen levels (Figure S2E; quantified in Figure S2F), as well as in cells within the matrix (Figure S2H, inset: compare frequently negatively stained cells on the surface of the matrix “Normoxia,” to darkly stained cells within the matrix “Hypoxia”). Moreover, to confirm the activity of TH-302 within the matrix, γH2AX staining was performed and demonstrated a significant upregulation in DNA damage response within the organotypic matrices for TH-302 and the combination therapy (Figure S2I). These data are consistent with the activity of TH-302 increasing within the lower oxygen environment of the organotypic matrix, where it plays a significant role in alleviating the overall hypoxia-induced resistance to AZD2014 (Figures 2F–2I).

### *In Vivo* Assessment of Subcutaneous PDAC Tumors Treated with AZD2014 in Combination with the HAP TH-302

**Figure 3:**
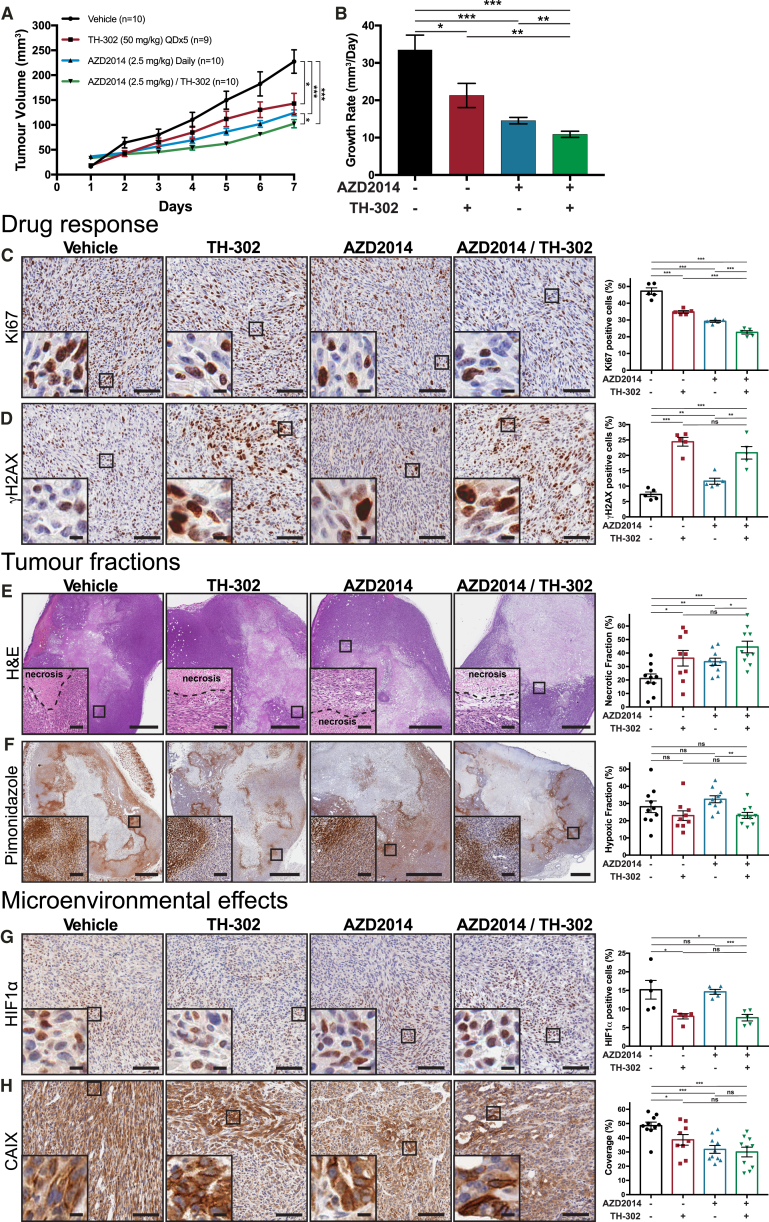
Assessment of KPC Subcutaneous Xenografts Treated with TH-302 (50 mg/kg) and AZD2014 (2.5 mg/kg) (A and B) Tumor volume measurements (A) and average linear growth rate (B) of tumors over 7 days from vehicle/saline (n = 10), vehicle/TH-302 (n = 9), AZD2014/saline (n = 10), and AZD2014/TH-302 (n = 10). (C and D) Immunohistochemistry (IHC) staining of drug response in tumors assessed for the proliferative marker Ki67 (n = 5 tumors/treatment) (C) and DNA damage response (γH2AX, n = 5 tumors/treatment) (D). Scale bars, 100 μm; insets, 10 μm. Mean ± SEM. (E and F) Staining for the necrotic (using H&E) (E) and hypoxic (pimonidazole IHC) (F) tumor fractions from vehicle/saline (n = 10), vehicle/TH-302 (n = 9), AZD2014/saline (n = 10), and AZD2014/TH-302 (n = 10) treatments. Scale bars, 1 mm; insets, 100 μm. Mean ± SEM. (G) HIF1α (n = 5 tumors/treatment) IHC staining of tumors. Scale bars, 100 μm; insets, 10 μm. Mean ± SEM. (H) Carbonic anhydrase IX (CAIX) IHC staining of tumors from vehicle/saline (n = 10), vehicle/TH-302 (n = 9), AZD2014/saline (n = 10), and AZD2014/TH-302 (n = 10) treatments. Scale bars, 100 μm; insets, 10 μm. Mean ± SEM. p values are from a Student two-tailed parametric t test in all panels. ^∗^p < 0.05, ^∗∗^p < 0.01, and ^∗∗∗^p < 0.001. See also Figure S3.

Guided by the spatial response of the combination therapy identified in our 3D organotypic matrices, we investigated whether this effect was recapitulated *in vivo*. KPC tumor-bearing mice were treated with a combination of AZD2014 (2.5 mg/kg) and TH-302 (50 mg/kg). Here, we observed a significant decrease in both tumor growth and Ki67 positivity within the combination-treated tumors, compared to AZD2014 monotherapy (Figures 3A–3C). Previous work has demonstrated that the DNA cross-linking effect of TH-302 is associated with an increase in γH2AX-positive cells, consistent with activation of the DNA damage response (Meng et al., 2012, Sun et al., 2015). In agreement, TH-302 treatment resulted in a significant increase in γH2AX-positive cells in this study (Figure 3D). Interestingly, AZD2014 alone also caused a significant increase in γH2AX-positive cells, in line with the known role of the PI3K pathway in sensitizing cells to DNA damage (Figure 3D) (Sun et al., 2015). Similarly, both the mono- and combination therapies showed an increase in necrosis, compared to vehicle treatment (Figure 3E). We concluded that both drugs were working as expected within the tumors, but the benefit of TH-302 combination therapy over AZD2014 monotherapy was not provided by an increased sensitization of cells to DNA damage.

We then assessed whether the reduced fraction of Ki67-positive cells between AZD2014 alone or in combination with TH-302 (Figure 3C) was due to a reduction in the hypoxic burden of the tumor. Assessing the hypoxic fraction of the tumors by pimonidazole staining, we observed a significant decrease between AZD2014 alone and the combination (Figure 3F, compare blue bar to green). This modest change seemed unlikely to be the only factor involved in the observed differences between the treatments. Moreover, it is important to note that pimonidazole is a marker of very low oxygen content and does not always coincide with the subtle changes in hypoxic response pathways within the cell (Figures S1G and S1H) (Varia et al., 1998, Wilson and Hay, 2011). To assess this more directly, we stained for HIF1α, which is responsible for mounting much of the cellular hypoxic response. This staining revealed a significant decrease in the fraction of cells actively responding to a low oxygen environment, in both TH-302 alone and combination treatments (Figure 3G). Furthermore, staining for carbonic anhydrase IX (CAIX), an important downstream component of the cellular pH-regulatory response in hypoxia (Parks et al., 2017), coincided with a significant decrease in CAIX expression in the mono- and combination therapies (Figure 3H). Additionally, to assess whether the derived benefit of the combination was a result of changes in vasculature, we quantified the coverage of microvessels stained for CD31 and found no significant difference between the treatments (Figure S3A).

**Figure 4:**
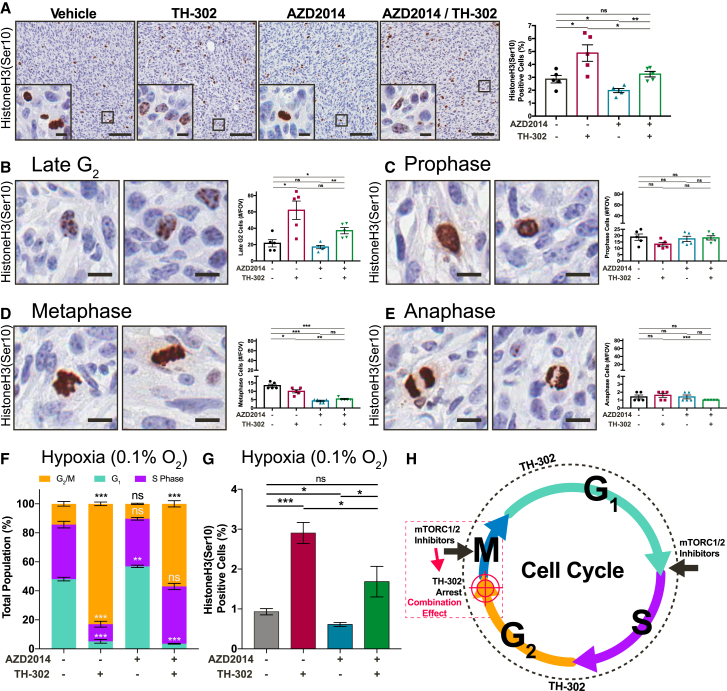
Characterization of the Cell Cycle Response of KPC Subcutaneous Xenografts Treated with TH-302 (50 mg/kg) and AZD2014 (2.5 mg/kg) (A) IHC staining of drug response in tumors assessed for the mitosis marker HistoneH3(Ser10) (n = 5 tumors/treatment). Scale bars, 100 μm; insets, 10 μm. Mean ± SEM. p values are from a Student two-tailed parametric t test. (B–E) Phenotypic quantification of four distinguishable stages of mitosis, namely late G_2_ (B), prophase (C), metaphase (D), and anaphase (E), where HistoneH3(Ser10) presents a unique phenotype. Scale bars, 10 μm. Mean ± SEM. p values are from a Student two-tailed parametric t test. (F) Propidium iodide staining of cell cycle phase distribution in KPC primary PDAC cells after treatment with AZD2014 (500 nM) and/or TH-302 (1 μM) at 0.1% oxygen. Mean ± SEM. p values were calculated using a two-way ANOVA with a Tukey correction for multiple comparisons. (G) HistoneH3(Ser10)/propidium iodide dual-parameter FACS analysis of KPC primary PDAC cells after treatment with AZD2014 (500 nM) and/or TH-302 (1 μM) at 0.1% oxygen. Mean ± SEM. p values are from a Student two-tailed parametric t test. (H) Schematic representation of the proposed combination effect of AZD2014 on cells prior to mitotic entry by TH-302. ^∗^p < 0.05, ^∗∗^p < 0.01, and ^∗∗∗^p < 0.001.

While these changes may be acting additively to elicit the growth inhibition and decreased Ki67 positivity in the combination therapy, we also assessed whether there was a combined effect of each treatment on the cell cycle. Initially, we scored the number of cells with fragmented nuclei in each condition and found a significant increase, which may indicate a higher rate of mitotic failure (Figure S3B). This led us to assess the effect of our therapies on mitotic progression, through HistoneH3(Ser10) staining (Figure 4A). By this approach, we found a significant upregulation of HistoneH3(Ser10)-positive cells in the TH-302 treatments and a significant downregulation in the AZD2014 treatments (Figure 4A). Upon further phenotypic assessment of the specific stages, from late G_2_ through to anaphase, we found that TH-302 causes cells to accumulate in late G_2_ at the initiation of mitosis, observing increased focal staining of HistoneH3(Ser10) within nuclei (Figures 4B–4E) (Hendzel et al., 1997). To confirm this effect *in vitro*, we performed FACS analysis of KPC cells treated with each inhibitor at 0.1% oxygen (Figure 4F). Here, we observed a significant arrest of TH-302-treated cells in G_2_/M, which we verified by observing HistoneH3(Ser10) accumulation (Figure 4G). TH-302 induces DNA damage in cells throughout the entire cell cycle, but our data suggest that in hypoxia TH-302-damaged cells arrest just prior to mitotic entry. This is likely to greatly enhance the mTORC1/2 inhibition of cells that escape this arrest (Figure 4H). Indeed, mTORC1 is independently regulated during mitosis and is required for efficient transit through the G_2_/M transition, and cytokinesis (Ramírez-Valle et al., 2010). Furthermore, mTORC2 has been shown to be essential for S and G_2_/M cell cycle progression after treatment with a DNA-damaging agent (Selvarajah et al., 2015). This may partially explain the additive effect found in the combination therapy (Figure 4H). These readouts however, do not provide any details with regard to the treatment-induced modulation of PI3K pathway activity, within both the hypoxic and normoxic tumor compartments. We therefore chose to track the hypoxic tumor regions, as an initial step toward mapping drug response.

### Mapping of Transient Hypoxic Tumor Regions *In Vivo*

**Figure 5:**
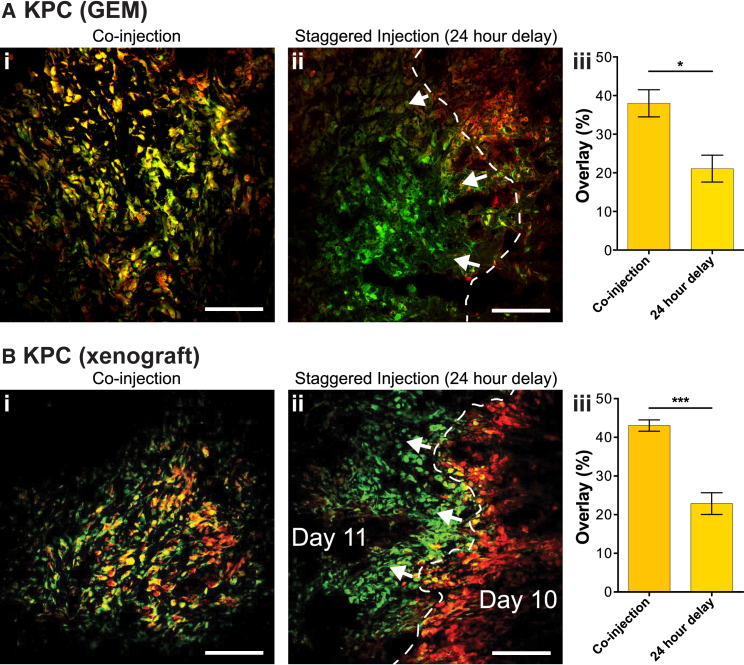
Tracking of Tumor Hypoxia with EF5 and Pimonidazole (A and B) Immunofluorescence of (A) KPC GEM tumors and (B) KPC xenograft tumors for EF5 (red) and pimonidazole (green), chemical indicators of tumor hypoxia, after either (i) co-injection or (ii) 24-hr delayed treatments. Scale bars, 100 μm. (iii) Quantification of overlapping (yellow) regions of staining between EF5 and pimonidazole in KPC GEM tumors (n = 3 mice/group) and KPC xenograft tumors (n = 4 mice/group). Mean ± SEM. p values are from a Student two-tailed parametric t test in all panels. See also Figure S4.

In order to map tumor hypoxia in more detail, we assessed the temporal change in the distribution of hypoxia over a 24-hr period. To track hypoxia, we employed both the GEM and subcutaneous KPC PDAC models, treating with the 2-nitroimidazole hypoxia markers EF5 (Lord et al., 1993) and pimonidazole (Varia et al., 1998). These markers were either co-injected or administered 24 hr apart. As expected, when the probes were co-injected, there was a strong co-localization of the two markers, with a large overlap of the immunofluorescence signals (Figure 5). Notably, when the EF5 and pimonidazole injections were staggered, 24 hr apart, we observed a distinct shift between the two markers and the overlap of the signals decreased (Figure 5). This effect was also observed in the KP^fl^C GEM model (Figure S4). Such a significant decrease in overlap in the staggered condition reveals that hypoxia moves transiently around the tumor. Many groups have described the important function of metabolic reprogramming over oxygen gradients, associated with the aberrant vascular networks that give rise to the formation of hypoxia in many cancers (Rodenhizer et al., 2016, Shukla et al., 2017). In such a rapid disease model, it then does not seem surprising that oxygen gradients should move around the tumor. This has important implications for cancer therapies, as the resistance that is associated with the hypoxic compartment may also transit between tumor regions. Fortunately, the use of HAPs, which become more active in these hypoxic regions, allows treatments to effectively hijack this phenomenon to release cytotoxics by a local bystander effect and potentially to reduce the hypoxia-induced resistance we have observed *in vivo*.

### Development of a Dual Imaging Modality for Parallel Monitoring of PI3K Pathway Activity and Tumor Oxygen Content in Live Cells

Having established the transient nature of the hypoxic gradients within the tumor, we then developed a method for live tracking of both drug response and oxygen content by real-time IVM. In contrast to static readouts, IVM provides a dynamic means to measure single-cell responses within their native microenvironment (Conway et al., 2014, Conway et al., 2017, Ellenbroek and van Rheenen, 2014). Initially, KPC cells were engineered to stably express the Eevee-Akt-mT2 FRET biosensor, which undergoes a conformational change in response to phosphorylation by Akt at a specific consensus sequence and so an increase in the FRET efficiency, which can be monitored using FLIM-FRET (Figure S5A) (Komatsu et al., 2011). Expression of this FRET biosensor caused no detectable change in the response of KPC cells to hypoxia, which also demonstrated a similar pattern of resistance for all three PI3K pathway inhibitors (Figures S5B–S5E). Akt is a key effector of the PI3K pathway and is inactivated by AZD2014 through mTORC2 inhibition (Figure S5F) (Driscoll et al., 2016). Since AZD2014 is weakly autofluorescent, we used an experimentally measured time-varying background (TVB) to account for background fluorescence from the cells and the drug (see Supplemental Experimental Procedures for details and validation) (Conway et al., 2017). Using FLIM-FRET imaging, we observed that initial activation of Akt by EGF was reduced by AZD2014, as expected (Figures S5G–S5J). In contrast, treatment with the mTORC1 inhibitor rapamycin had no inhibitory effect on Akt. This is in line with previous reports, where inhibition of mTORC1 alone instead acts to upregulate Akt activity through IRS1 (O’Reilly et al., 2006). Having established the specificity of the Eevee-Akt-mT2 FRET biosensor and the significant response of the biosensor-expressing cells to treatment with AZD2014, we next developed a method for parallel monitoring of oxygen content by dual FLIM/PLIM imaging.

**Figure 6:**
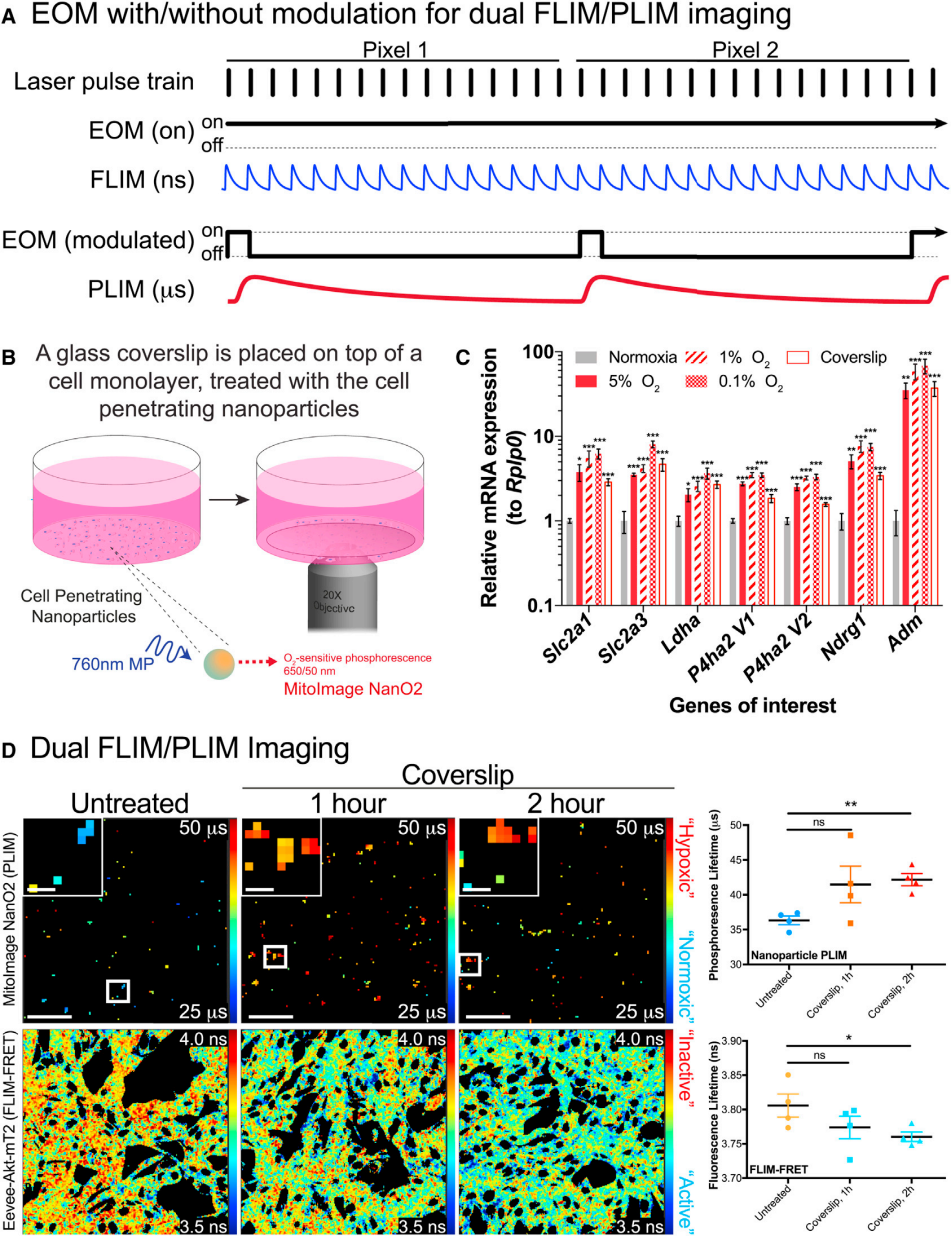
Dual FLIM/PLIM Imaging of KPC Cells Stably Expressing the Eevee-Akt-mT2 Intramolecular FRET Biosensor, Treated with Oxygen-Sensitive Nanoparticles (A) A schematic illustration of the methodology applied to modify the multiphoton FLIM detection system to detect oxygen-sensitive PLIM through modulation of the EOM. (B) A schematic demonstrating the use of a glass coverslip to induce a hypoxic response *in vitro*. (C) qRT-PCR analysis of relative mRNA expression of hypoxia response genes upregulated in cells incubated for 2 hr in hypoxia (5%, 1%, and 0.1% oxygen) or under a coverslip, compared to normoxia, normalized to *Rplp0* (n = 5). Mean ± SEM. (D) Dual FLIM/PLIM imaging of Akt activity in KPC cells treated with MitoImage NanO2 and incubated with a glass coverslip for 1 or 2 hr (n = 4). Scale bars, 50 μm; insets, 10 μm. Mean ± SEM. p values are from a Student two-tailed parametric t test in all panels. ^∗^p < 0.05, ^∗∗^p < 0.01, and ^∗∗∗^p < 0.001. See also Figures S5 and S6, and Tables S1 and S2.

The first step toward our dual imaging modality was to assess the effect of low oxygen levels on the activity of Akt by FLIM-FRET microscopy, and in line with the western blot assessment (Figures 1D and S5B), we found a significant increase in Akt activity in hypoxia (1% and 0.1% oxygen) compared to normoxia (Figure S6A). An emerging option for live imaging of tumor oxygen content is through the use of oxygen-sensitive ruthenium, platinum, or iridium complexes as nanoparticles (Conway et al., 2017, Kalinina et al., 2016, Kondrashina et al., 2012, Zheng et al., 2015). Recently, imaging of the intrinsic autofluorescence of NAD(P)H/FAD^+^ was combined with PLIM of an oxygen-sensitive ruthenium complex (Kalinina et al., 2016). This approach used on-off modulation of the pulsed laser source on a microsecond timescale to allow measurements of the longer oxygen-sensitive phosphorescence decays (Kalinina et al., 2016). Here, we took a similar approach, using an electro-optic modulator (EOM) to switch a multiphoton laser at low frequency (Figure 6A). This allowed us to detect both the short FLIM decays (nanosecond scale) from the Eevee-Akt-mT2 biosensor, and the much longer phosphorescence decays (microsecond scale) of an oxygen-sensitive nanoparticle (Figure 6A).

In order to validate our approach *in vitro*, we placed glass coverslips over KPC cell monolayers to reduce the oxygen concentration (Figure 6B). First, however, we characterized the application of this coverslip hypoxia induction by staining cells for nuclear HIF1α stabilization and pimonidazole adduct formation, and observed a significant increase in both after 2 hr, equivalent to 0.1% or 1% oxygen incubations (Figures 6B and S6B; quantified in Figures S6C and S6D). Furthermore, we assessed the response of cells under a coverslip by quantitative real-time PCR (qRT-PCR) for genes characteristically upregulated under hypoxic conditions and found a significant upregulation of *Slc2a1* (GLUT1), *Slc2a3* (GLUT3), *Ldha* (lactate dehydrogenase A), *P4ha2 V1/V2* (prolyl 4-hydroxylase), *Ndrg1*, and *Adm* (adrenomedullin) (Figure 6C) (Miller et al., 2015, Shukla et al., 2017). In this way, we could confirm that a 2-hr incubation with a glass coverslip was sufficient to decrease oxygen levels and relieve the oxygen-dependent quenching of MitoImage NanO2, which led to a significant increase in the PLIM (Figure 6D, top panel; note shift in PLIM from blue to orange/red upon decrease in oxygen content). In parallel, we were able to track the activity of Akt in the same cells by FLIM-FRET microscopy of the Eevee-Akt-mT2 intramolecular FRET biosensor and observed a significant increase in the FRET efficiency, consistent with an increase in Akt activity (Figure 6D, bottom panel; note shift in FLIM from orange to blue). As an additional confirmation, we treated parental KPC cells with MitoImage MM2, which is composed of an oxygen-sensitive phosphorescent (PtTFPP) and oxygen-insensitive fluorescent (PFO) component that can be read out ratiometrically, to assess oxygen content (Kondrashina et al., 2012). By placing a glass coverslip on these cell monolayers, we observed a significant increase in the ratio of the oxygen-sensitive PtTFPP to the oxygen-insensitive PFO, consistent with a decrease in the oxygen content of the cell monolayer (Figure S6E).

### Live Tracking of Tumor Oxygen Content Allows Real-Time Assessment of Drug Response by IVM

**Figure 7:**
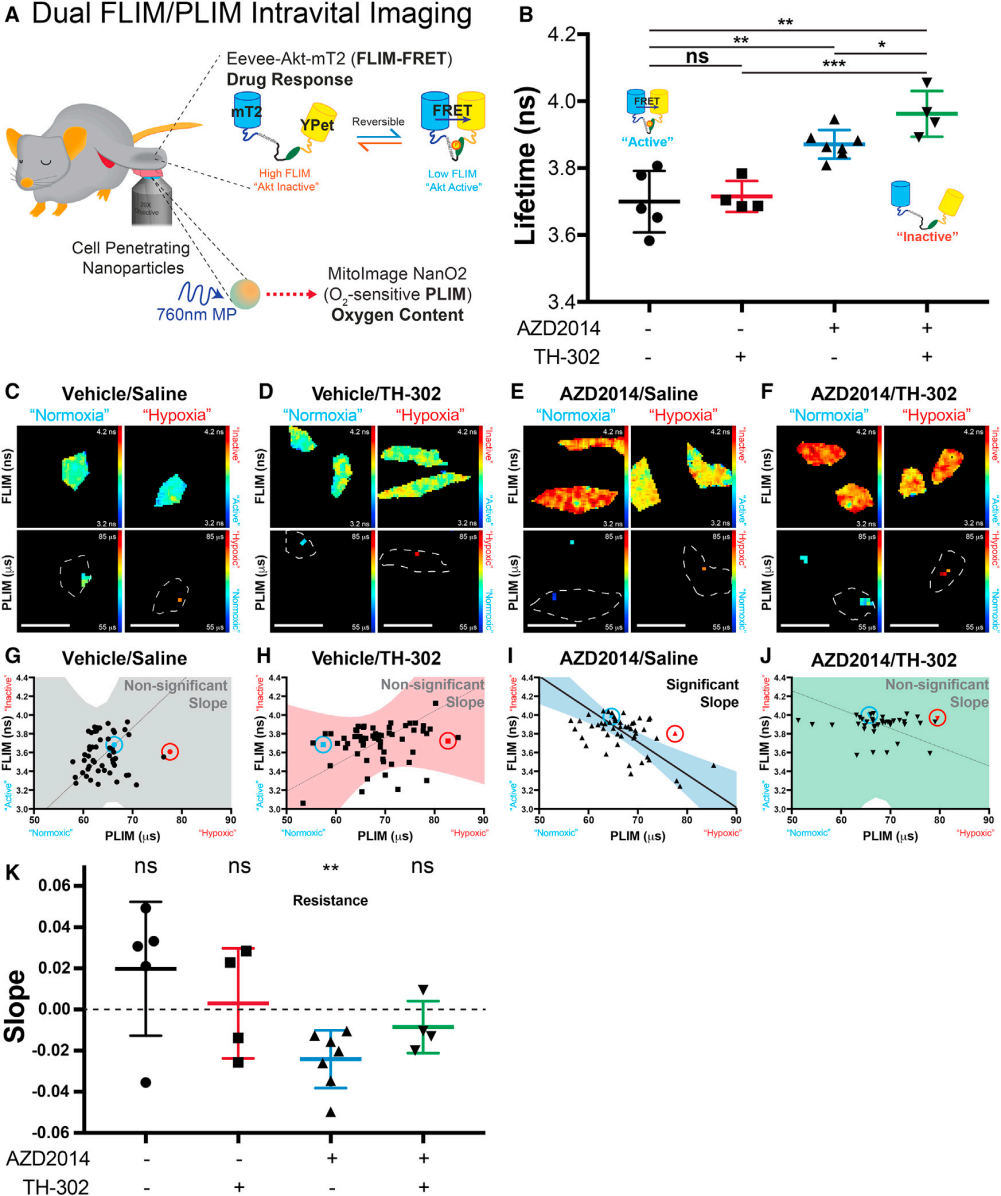
Dual FLIM/PLIM Intravital Imaging of Drug Response and Tumor Oxygen Content (A) A schematic representation of the intravital imaging setup for the dual FLIM/PLIM imaging. Xenografts of KPC cells stably expressing the Eevee-Akt-mT2 intramolecular FRET biosensor were allowed to reach 350 mm^3^, before treatment with oxygen-sensitive nanoparticles (NanO2) and dual FLIM/PLIM imaging. (B) FLIM-FRET analysis of vehicle/saline (n = 5), vehicle/TH-302 (n = 4), AZD2014/saline (n = 7), or AZD2014/TH-302 (n = 4)-treated mice. Mean ± SEM. p values are from a Student two-tailed parametric t test. (C–F) Representative FLIM and PLIM maps are provided for cells with a short PLIM value (high oxygen content, normoxic) and a long PLIM value (low oxygen content, hypoxic) for vehicle/saline (C), vehicle/TH-302 (D), AZD2014/saline (E), and AZD2014/TH-302 (F) treatments. Scale bars, 25 μm. (G–J) These same cells are then highlighted on their representative Deming regression curves, where blue and red points highlight cells with short or long PLIM values, respectively. The 95% confidence intervals emphasize whether the slope of the Deming regression is significantly non-zero for vehicle/saline (G), vehicle/TH-302 (H), AZD2014/saline (I), and AZD2014/TH-302 (J) treatments. (K) Average Deming slopes of mice from each treatment group, assessed for departure from zero with a one-sample t test. Mean ± SEM. ^∗^p < 0.05, ^∗∗^p < 0.01 and ^∗∗∗^p < 0.001.

Having established the dual FLIM/PLIM imaging methodology for *in vitro* imaging, we next assessed the effect of the combination therapy on Akt activity in a live setting. Consistent with the tumor growth experiment (Figure 3), mice were given AZD2014 and TH-302, and once subcutaneous xenografts reached a volume of >350 mm^3^, where hypoxia is readily observed, tumors were surgically exposed and treated with the oxygen-sensitive nanoparticles (Figure 7A) (Conway et al., 2017, Vennin et al., 2017). Assessing single cells for their response to each treatment, we found no significant difference in Akt activity between vehicle and TH-302 treatments, as expected (Figure 7B, compare black plot to red). Conversely, upon treatment with AZD2014, a significant decrease in Akt activity was recorded (Figure 7B, compare black plot to blue). Furthermore, when AZD2014 was used in combination with TH-302, Akt activity was significantly reduced to a greater degree than AZD2014 monotherapy (Figure 7B, compare blue plot to green). This effect was then stratified based on the oxygen content by analyzing the Akt activity of individual cells (FLIM-FRET) and the oxygen content by PLIM (Figures 7C–7F, top panel, Akt activity [FLIM-FRET]; bottom panel, oxygen content [PLIM]). In this way, we observed heterogeneity in Akt activity for vehicle and TH-302 treatments, as expected (Figures 7C and 7D; plotted in Figures 7G and 7H, respectively). However, we found that cells treated with AZD2014 monotherapy had reduced Akt activity predominantly in the high-oxygen (normoxia) regions and less so in the low-oxygen (hypoxia) regions (Figure 7E; plotted in Figure 7I). Importantly, this effect was absent in the combination therapy, where Akt activity was reduced in both high- and low-oxygen regions (Figure 7F; plotted in Figure 7J). This confirms and builds upon our *in vitro* findings on hypoxia-induced resistance, prompting further analysis of the *in vivo* effect of oxygen content on drug response.

In order to quantify the effect of oxygen content on drug response, we plotted the fluorescence lifetime from the Eevee-Akt-mT2 FRET biosensor for Akt activity (FLIM-FRET), against the phosphorescence lifetime of MitoImage NanO2, as a measure of oxygen content (PLIM; Figures 7G–7J). Using Deming regression curves, to account for the error in both parameters (FLIM-FRET and PLIM), Deming slopes were calculated and plotted with their 95% confidence bands to highlight the quality of the fit (Figures 7G–7J). For the vehicle treatment, the Deming regression slope was not significantly different from zero, which meant that the error in the slope was sufficiently large and that the confidence in the slope did not exist (Figure 7G). This was consistent with the heterogeneous Akt activity in both the vehicle and TH-302-treated mice (Figure 7K, see black and red bars). In the AZD2014 treatment, however, the slope of the regression was negative and significantly non-zero (Figure 7K, see blue bar). This identified a significant link between increased Akt activity and low oxygen content, demonstrating that, in the AZD2014 treatments, there was hypoxia-induced resistance (Figure 7I). Cumulative assessment of the AZD2014 treatments confirmed this negative slope was significant, indicating the presence of hypoxia-induced resistance to AZD2014 monotherapy within our live tumors (Figure 7K). Furthermore, when AZD2014 treatment was combined with TH-302, this resistance was relieved (Figure 7K, see green plots), consistent with the improved inhibition of Akt for the combination treatments (Figure 7B). In this way, we have identified hypoxia-induced resistance to PI3K pathway targeting, at the single-cell level, within our live tumors by IVM and successfully alleviated this *in vivo* resistance in combination with the HAP TH-302.

## Discussion

Increasingly, our awareness of the context-dependent effects on treatment efficacy, such as microenvironmental factors and the influence of tumor-associated immune or stromal components, is driving the development of more sophisticated IVM approaches to account for these important contributing factors (Conway et al., 2017, Ellenbroek and van Rheenen, 2014, Junankar et al., 2015, Vennin et al., 2017). PDAC has a well-established hypoxic signature, and this hypoxia was clearly evident in our GEM and subcutaneous mouse models (Chang et al., 2011, Miller et al., 2015). Moreover, a hypoxic tumor microenvironment is strongly associated with treatment-refractory disease (Sullivan et al., 2008, Wilson and Hay, 2011), and here we demonstrate hypoxia-induced resistance to three PI3K pathway inhibitors. This occurs in parallel with an increase in the activity of Akt, a key effector of the PI3K pathway. In light of our recent work, demonstrating an equivalent survival benefit for AZD2014, a dual inhibitor of the PI3K pathway, with standard-of-care gemcitabine (Driscoll et al., 2016), these data provide strong support for investigation into the effects of hypoxia on PI3K pathway targeting.

Given our initial insight into the persistent movement of hypoxic regions throughout the tumor, the ability to track and target this key microenvironmental factor has a clear benefit for pre-clinical investigations aimed at negating the effects of tumor hypoxia. However, few methods exist for live monitoring of hypoxia by IVM and they lack the parallel approach that would be necessary to investigate the effects of hypoxia on therapeutic response (Conway et al., 2017). For whole-tumor assessment, many non-invasive positron emission tomography (PET)-based approaches depend upon the increased glucose uptake of tumors (Fleming et al., 2015, Rajendran et al., 2004). Of note, new probes have been developed to provide information on the hypoxic content of tumors, based on CAIX expression or 2-nitroimidazoles, primarily to guide hypoxia-targeted treatment strategies (Fleming et al., 2015, Hoeben et al., 2010, Rajendran et al., 2004). However, for molecular assessment during preclinical drug development, a new approach was necessary to overlay tumor oxygen content with therapeutic response by IVM. On the one hand, genetic markers based on the oxygen degradation domain or hypoxia-response elements of HIF1α are common (Erapaneedi et al., 2016, Wang et al., 2016), but these may encounter hurdles for interpretation due to the dysregulation of the hypoxic response in many cancers and the necessity to measure changes in intensity, instead of a switch-like response. On the other hand, many injectable fluorescent probes exist for tracking various aspects of tumor hypoxia (O’Connor et al., 2017, Uddin et al., 2015, Zheng et al., 2015). In this work, application of oxygen-sensitive nanoparticles for dual FLIM/PLIM imaging facilitated live tracking of drug response in the context of tumor oxygen content. Critically, we identified an oxygen-dependent resistance effect, at the single-cell level, when treating tumors with AZD2014 monotherapy. This effect was reduced *in vivo*, when AZD2014 was combined with the HAP TH-302, leading to improved inhibition of tumor growth. TH-302 alone, or in combination with AZD2014, resulted in a decreased hypoxic fraction, HIF1α and CAIX expression. This reduction in tumor hypoxic response was associated with a concomitant reduction in overall resistance to PI3K pathway targeting and provides a possible explanation for the improved reduction in Akt activity in the combination therapies.

Preclinical IVM experiments can provide a robust method to assess combination therapies aimed at reducing resistant populations and improving therapeutic responses. This possibility has yet to be fully exploited for the known hypoxia-induced resistance to targeted therapeutics aimed at tyrosine kinase, HER2, and VEGF pathways (Ahmadi et al., 2014, Bergers and Hanahan, 2008, Karakashev and Reginato, 2015), as well as chemotherapies (Shukla et al., 2017, Sullivan et al., 2008), and highlights the clinical need to track and understand this hypoxia-induced resistance. Intramolecular FRET biosensors already exist to read out the activity of many of these pathways, while the development of simplified backbones for fluorescent protein reporters or FRET biosensors provides a rapid approach to develop readouts for therapeutic response within a live tumor context (Conway et al., 2014, Conway et al., 2017, Komatsu et al., 2011). As demonstrated here and elsewhere, the addition of HAPs as a combination therapy provides a suitable approach to reduce the resistant effects of hypoxia with reduced additive toxicity (O’Connor et al., 2016, Wilson and Hay, 2011). By applying IVM using fluorescent protein reporters or FRET biosensors and parallel imaging of tumor hypoxia, the benefits observed here for targeting of the PI3K pathway, in combination with HAPs, can be applied to develop combination therapies with robust preclinical validation.

## Experimental Procedures

### Animal Experiments

All animal experiments were conducted in compliance with Garvan Ethics Committee guidelines (13/17 and 16/13 protocols) and in accordance with the Australian code of practice for the care and use of animals for scientific purposes, with genotyping performed by Garvan Molecular Genetics (Sydney, NSW, Australia). Both male and female KP^fl^C and KPC GEM were monitored for swollen abdomen, cachexia, and reduced mobility until tumor was evident by palpation, and hypoxia markers were administered (Hingorani et al., 2003, Hingorani et al., 2005, Morton et al., 2010). For xenografts experiments, 1 × 10^6^ KPC primary PDAC cells in PBS were subcutaneously injected into the rear flank of 6- to 10-week-old female BALB/c-Fox1nuAusb mice.

### FACS

Cells were fixed in 70% ethanol, prior to staining with propidium iodide (Sigma-Aldrich; P4170; 1 μg/mL) with RNaseA (Sigma-Aldrich; R6513; 500 μg/mL), and analysis on a FACSCanto II (BD Biosciences). Quantification was performed in FlowJo (Tree Star), and cell cycle phase was determined in ModFit (Verity Software House).

### Intravital FLIM/PLIM Imaging

Xenografts of KPC cells expressing the Eevee-Akt-mT2 FRET biosensor were imaged at a final volume of 350 mm^3^, as per the tumor growth studies. Mice were anesthetized with xylazine (10 mg/kg) and Zoletil (50 mg/kg) and kept on a heated stage at 37°C. Anesthesia was maintained with isoflurane (3 L; O_2_, 1 L; vacuum, 1 L/min). Tumors were surgically exposed, as described previously (Conway et al., 2017, Vennin et al., 2017), prior to application of MitoImage NanO2 (10 μg; Ibidi; 74151). After 30 min, dual FLIM/PLIM imaging was performed on a Leica SP8 microscope with a 0.95 numerical aperture (NA), 25× water objective. Analysis of FLIM and PLIM decays was performed in FLIMfit (Warren et al., 2013) and is presented as mean lifetime per cell.

### Organotypic Assay

Organotypic matrices were generated as described previously (Conway et al., 2014, Morton et al., 2010, Vennin et al., 2017). Quantification of the invasive and proliferative indices was performed on pan-cytokeratin (excludes fibroblasts) or Ki67-stained sections, respectively.

### IC_50_ Curves

IC_50_ curves were performed in a 96-well plate format, with 1,000 cells/well. Cells were incubated for 24 hr after seeding before addition of inhibitors. Plates were assessed for relative cell density using the CellTiter 96 AQueous Cell Proliferation Assay (Promega; G1111) at 72 hr after addition of each inhibitor, with parallel plates incubated under standard normoxic or hypoxic (0.1% oxygen) conditions. IC_50_ curves were then fit in Prism (GraphPad Software), normalizing to the vehicle (DMSO) control treatments from the respective normoxic or hypoxic (0.1% oxygen) conditions.

### Statistical Analysis

Statistical tests were performed in Prism (GraphPad Software) with statistical significance given as ^∗^p < 0.05, ^∗∗^p < 0.01, and ^∗∗∗^p < 0.001 in all cases. A one-sample t test was performed on normalized data. For IC_50_ curve comparisons, an extra sum-of-squares F test was performed between the best-fit parameters of each curve. Deming regression curves for the dual FLIM/PLIM analysis were plotted with the “mcr” package of R, using the analytical method to calculate the 95% confidence intervals. To assess whether the Deming regression slopes were significantly non-zero, a one-sample t test was performed. DNA cell cycle analysis was assessed for significance using a two-way ANOVA test with a Tukey correction for multiple comparisons. In all other cases, a Student two-tailed parametric t test was performed.

Detailed protocols can be found in Supplemental Experimental Procedures.

## Supplemental Information

Document S1. Supplemental Experimental Procedures, Figures S1–S6, and Tables S1 and S2

Document S2. Article plus Supplemental Information
