# Supplementary material for: Intravital Imaging to Monitor Therapeutic Response in Moving Hypoxic Regions Resistant to PI3K Pathway Targeting in Pancreatic Cancer
Source: Cell Rep. 2018 Jun 13;23(11):3312–26. doi: 10.1016/j.celrep.2018.05.038 (PMC6019737; doi:10.1016/j.celrep.2018.05.038)
Supplement: Document S1. Supplemental Experimental Procedures, Figures S1–S6, and Tables S1 and S2 [file mmc1.pdf]

Cell Reports, Volume *23*

Supplemental Information

Intravital Imaging to Monitor Therapeutic Response in Moving Hypoxic Regions Resistant to PI3K Pathway Targeting in Pancreatic Cancer

James R.W. Conway, Sean C. Warren, David Herrmann, Kendelle J. Murphy, Aurélie S. Cazet, Claire Vennin, Robert F. Shearer, Monica J. Killen, Astrid Magenau, Pauline Mélénec, Mark Pinese, Max Nobis, Anaiis Zaratzian, Alice Boulghourjian, Andrew M. Da Silva, Gonzalo del Monte-Nieto, Arne S.A. Adam, Richard P. Harvey, Jody J. Haigh, Yingxiao Wang, David R. Croucher, Owen J. Sansom, Marina Pajic, C. Elizabeth Caldon, Jennifer P. Morton, and Paul Timpson

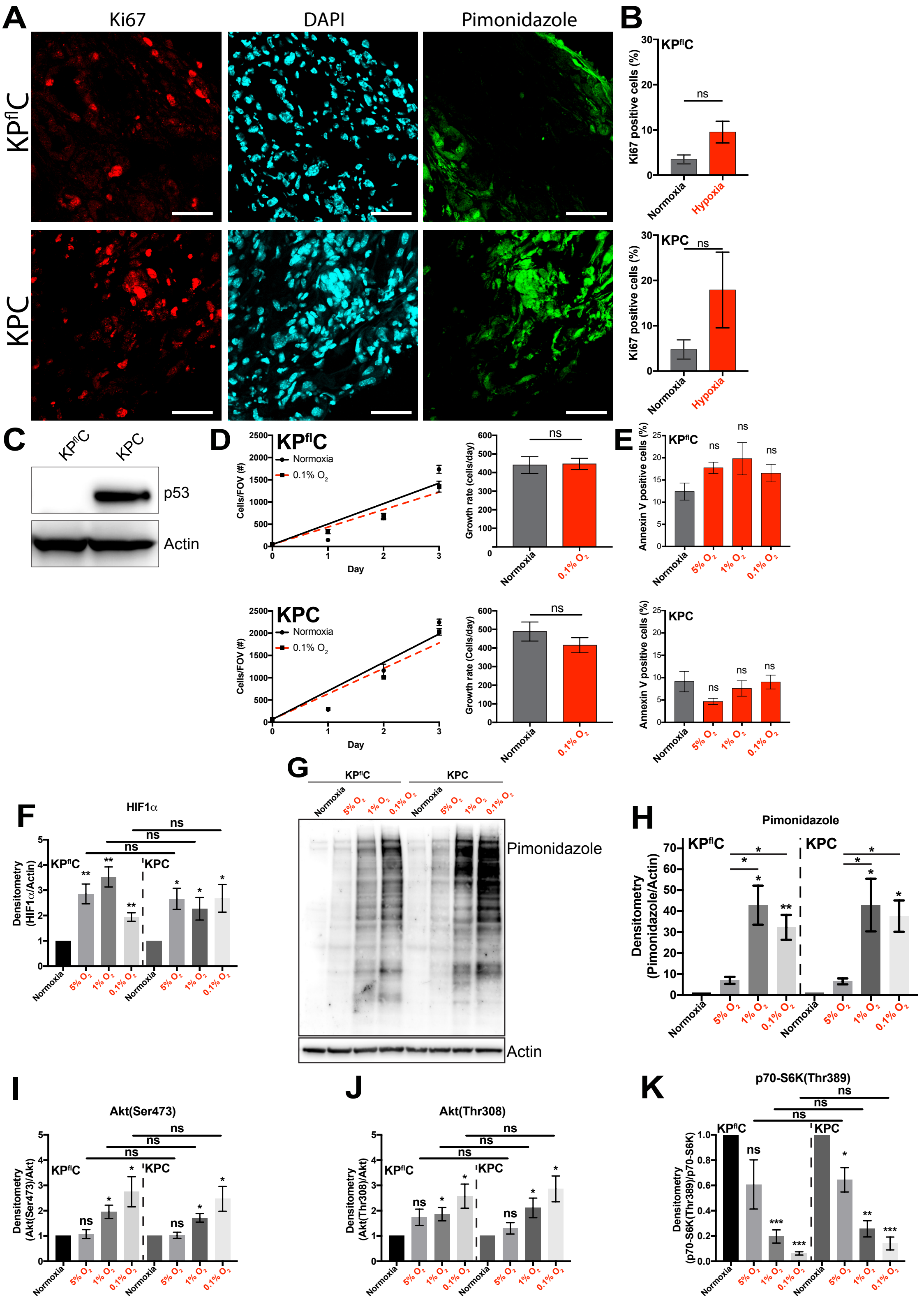

Figure S1, related to Figure 1. The presence of hypoxia and the associated growth and molecular effects in the KP[fl]C and KPC mouse models of PDAC. (A) Immunofluorescence staining of the GEM KP[fl]C and KPC PDAC mouse models for Ki67 (red), DAPI (cyan) and pimonidazole (green). Scale bars: 50 µm. (B) Quantification of Ki67 positive nuclei as a fraction of total nuclei, in pimonidazole negative (normoxic) and positive (hypoxic) regions (n=5 tumours/mouse model). Mean ± SEM. A one-sample t-test was performed on normalized data. (C) Representative western blot of p53 loss or gain-of-function mutant expression in the KP[fl]C and KPC primary PDAC cell lines respectively, after 48 hours of culture in normoxia (n=5). P values are from a Student's two-tailed parametric t-test. (D)

Assessment of growth rate in normoxic and hypoxic (0.1% oxygen) conditions in the $KP^{fl}C$ and KPC cell lines, over a 3 day time course (n=5). Mean ± SEM. (E) Quantification of Annexin V positive $KP^{fl}C$ and KPC cells by FACS analysis, after incubation for 48 hours in normoxia or hypoxia (5%, 1% and 0.1% oxygen). Mean ± SEM. P values are from a Student's two-tailed parametric t-test. (F) Densitometry of HIF1α western blot in Figure 1D (n=5). Mean ± SEM. A one-sample t-test was performed on normalized data. (G) Representative western blot of pimonidazole adducts in the $KP^{fl}C$ and KPC primary PDAC cell lines after 48 hours of culture in normoxia and 2 hours treatment with pimonidazole (236 μM, n=5). (H) Densitometry of pimonidazole western blot (n=5). Mean ± SEM. (I-K) Densitometry of Akt(Ser473), Akt(Thr308) and p70-S6K(Thr389) western blots in Figure 1D (n=5). Mean ± SEM. A one-sample t-test was performed on normalized data. *p<0.05, **p<0.01 and ***p<0.001.

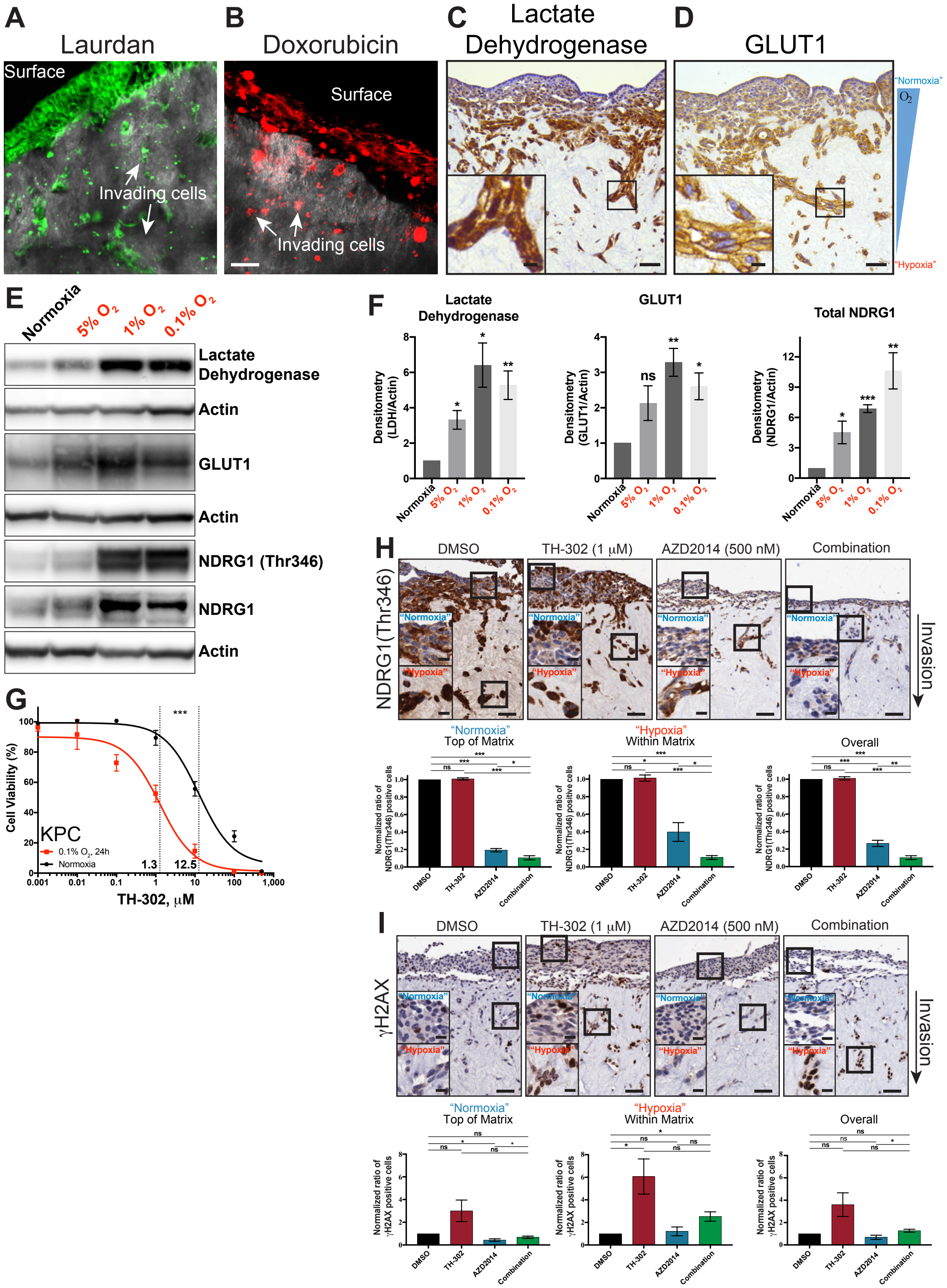

Figure S2, related to Figure 2. Assessment of hypoxia within organotypic matrices, as well as drug penetrance and response. Representative images of (A) laurdan (green; 2.5 μM) and (B) doxorubicin (red; 1 μM) treated organotypic matrices with invading KPC cells (14 day invasion), after 2 hours with the compound (n=3). SHG of the organotypic collagen matrix is shown as grey on the image. Scale bars: 50 μm. (C,D) Representative immunohistochemistry (IHC) images of lactate dehydrogenase and GLUT1 stained organotypic matrices with invading KPC cells (14 day invasion; n=4). Scale bars: 50 μm, scale bars (inset): 10 μm. (E) Representative western blot of the KPC primary PDAC cell line, incubated for 48 hours in normoxia or hypoxia (5%, 1% or 0.1% oxygen; n=5). (F) Densitometry of lactate dehydrogenase, GLUT1 and NDRG1(Thr346) western blots (n=5). Mean ± SEM. (G) IC_{50} curve of KPC cells treated with the HAP TH-302 in both normoxic (black lines) and hypoxic (0.1%

oxygen, red lines) conditions (n=3). An extra sum-of-squares F test was performed between the best-fit parameters of each curve. (H,I) Representative images of invading KPC cells stained with NDRG1(Thr346) or γH2AX, with quantification of the percentage of positively stained KPC cells on the surface ("Normoxia") or invading into ("Hypoxia") the organotypic matrices (n=4). Scale bars: 50 µm, scale bars (insets): 10 µm. Mean ± SEM. A one-sample t-test was performed on all normalized data. *$p<0.05$, **$p<0.01$ and ***$p<0.001$.

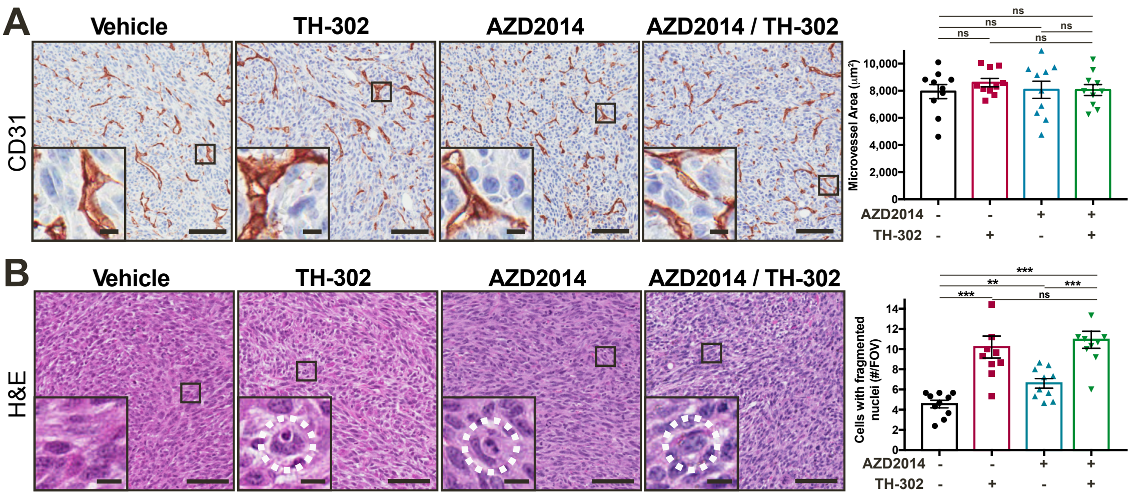

Figure S3, related to Figure 3. IHC staining of tumours for (A) the vascular marker CD31 and (B) the number of cells with fragmented nuclei per field-of-view (FOV) for vehicle/saline (n=10), vehicle/TH-302 (n=9), AZD2014/saline (n=10) and AZD2014/TH-302 (n=10). Scale bars: 100 µm, scale bars (insets): 10 µm. Mean ± SEM. P values are from a Student's two-tailed parametric t-test in all panels. *p<0.05, **p<0.01 and ***p<0.001.

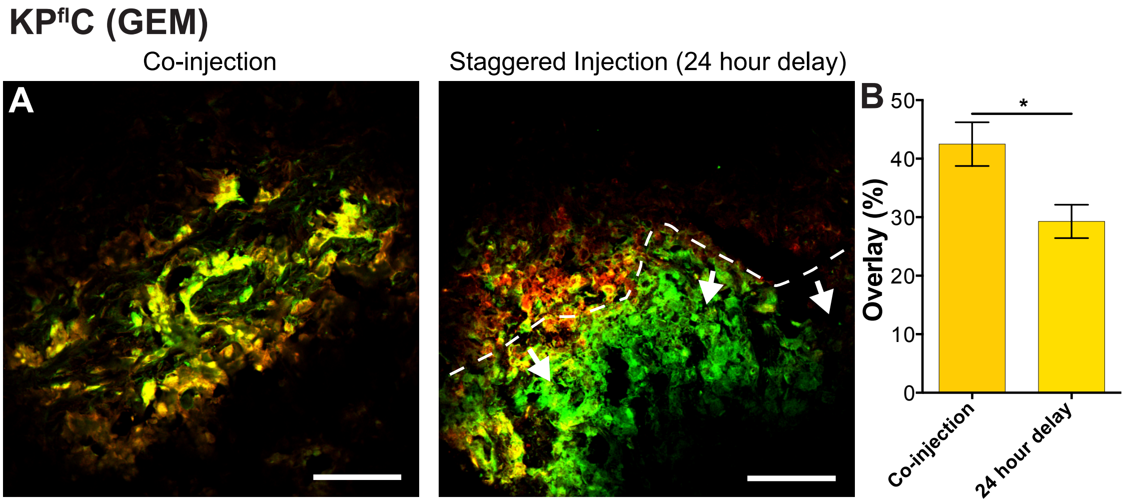

Figure S4, related to Figure 5. Tracking of tumour hypoxia with EF5 and pimonidazole. (A) Immunofluorescence of $KP^{fl}C$ GEM tumours for EF5 (red) and pimonidazole (green), chemical indicators of tumour hypoxia, after either co-injection or 24 hour delayed treatments. (B) Quantification of overlapping (yellow) regions of staining between EF5 and pimonidazole (n=3 mice/group). Scale bars: 100 µm. Mean ± SEM. The P value is from a Student's two-tailed parametric t-test. *p<0.05, **p<0.01 and ***p<0.001.

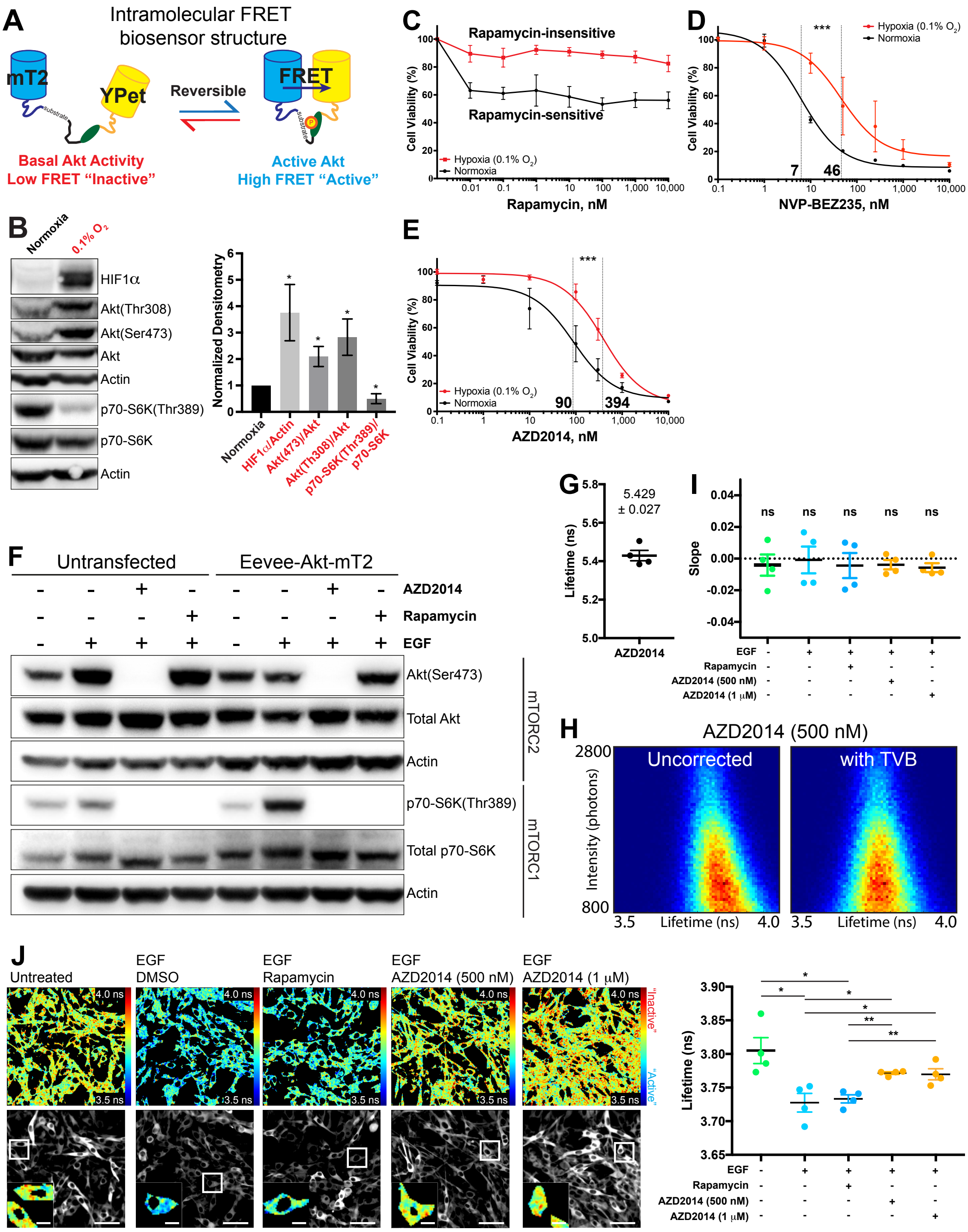

Figure S5, related to Figure 6. Characterization of KPC cells expressing an intramolecular FRET biosensor for Akt activity. (A) Schematic representation of the Eevee-Akt-mT2 FRET biosensor, which is composed of a FRET pair, namely mTurquoise2 (mT2) and YPet, a consensus substrate sequence for Akt (RKRDRLGTLGD), an Eevee linker (Komatsu et al., 2011) and a phosphorylation-binding domain from the yeast Rad53 (FHA1). (B) Representative western blots and densitometry of KPC cells stably expressing the Eevee-Akt-mT2 intramolecular FRET biosensor in both normoxic and hypoxic (0.1% oxygen) conditions (n=6). Mean ± SEM. A one-sample t-test was performed on normalized data. (C-E) IC_{50} curves of Eevee-Akt-mT2 expressing KPC cells treated with the PI3K-pathway targeted therapeutics, rapamycin, NVP-BEZ235 and AZD2014, in both normoxic (black lines) and hypoxic (0.1% oxygen, red lines) conditions (n=3). An extra sum-of-squares F test was performed between the best-fit parameters of each curve. (F) Representative western blots of AZD2014 and rapamycin treated KPC cells, with or without stable expression of the Eevee-Akt-mT2 intramolecular FRET biosensor (n=3). (G) Assessment of the autofluorescence lifetime of AZD2014 (2 μM) in untransfected KPC

cells. Mean ± SEM. (H) Representative intensity-lifetime correlation plots of AZD2014 (500 nM) treated KPC cells expressing the Eevee-Akt-mT2 FRET biosensor, with and without a time-varying background (TVB). (H) Average slopes from linear regression curves plotting the intensity of each cell against its' lifetime in each treatment, after correction with a TVB, assessed for departure from zero with a one-sample t-test. Mean ± SEM. (J) Representative fluorescence lifetime maps, corresponding intensity images and quantification of Eevee-Akt-mT2 expressing KPC cells treated with EGF (20 ng/ml), then either AZD2014 (500 nM or 1 μM) or rapamycin (100 nM; n=4). Scale bars: 50 μm, scale bars (insets): 10 μm. Mean ± SEM. P values are from a Student's two-tailed parametric t-test. *p<0.05, **p<0.01 and ***p<0.001.

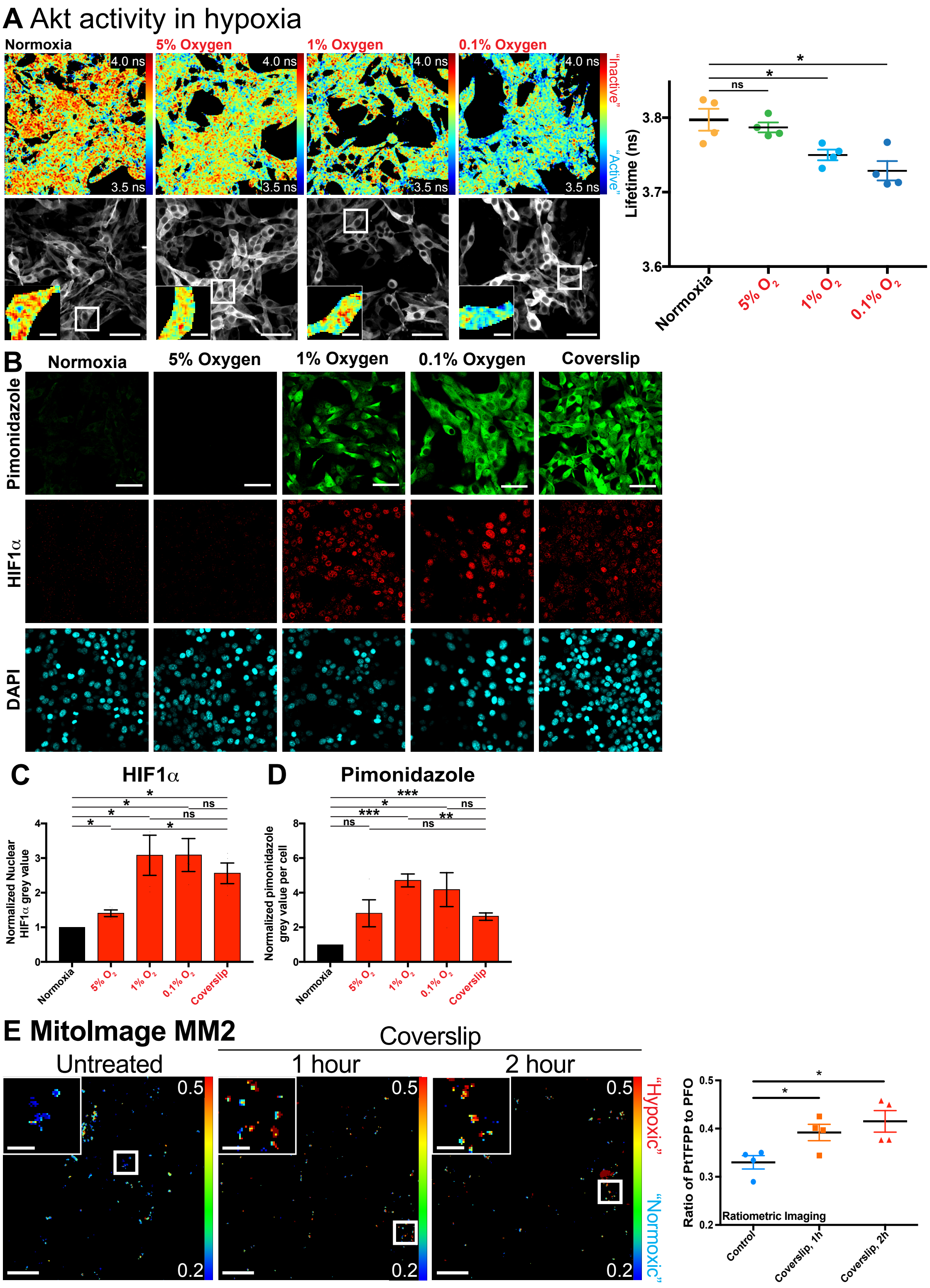

Figure S6, related to Figure 6. Characterization of hypoxia induction resultant from a cover glass on top of a KPC cell monolayer. (A) Representative fluorescence lifetime maps, corresponding intensity images and quantification of Eevee-Akt-mT2 expressing KPC cells incubated in normoxia or hypoxia (5%, 1% and 0.1% oxygen) for 2 hours prior to fixation and imaging (n=4). Scale bars: 50 µm, scale bars (insets): 10 µm. Mean ± SEM. (B) Representative immunofluorescence images of KPC cells stained for pimonidazole, HIF1α and DAPI after incubation in normoxia, hypoxia (5%, 1% and 0.1% oxygen) or under a coverslip for 2 hours. Quantification of the mean grey value/cell of (C) HIF1α and (D) pimonidazole in each treatment (n=4). Mean ± SEM. (E) Ratiometric imaging of KPC cells treated with oxygen-sensitive nanoparticles (MitoImage MM2) after incubation for 1 or 2 hours with a glass

coverslip (n=4). Scale bars: 50 µm, scale bars (insets): 10 µm. Mean ± SEM. Normalized data was assessed by a one-sample t-test. All other P values are from a Student's two-tailed parametric t-test. $^*p<0.05$, $^{**}p<0.01$ and $^{***}p<0.001$.

Table S1, related to Figure 6. List of qRT-PCR probes

Gene	Roche Universal Probe Library System		
	Forward Primer	Reverse Primer	UPL Probe
Slc2a1	GAC CCT GCA CCT CAT TGG	GAT GCT CAG ATA GGA CAT CCA AG	99
Slc2a3	TTT GCC CTG AGA GTC CAA GA	ACA AGC GCT GCA GGA TCT	69
Ldha	GGC ACT GAC GCA GAC AAG	TGA TCA CCT CGT AGG CAC TG	12
P4ha2 V1	TGT ATT CTG GTA CAA CCT TCT TCG	GGA ACC ACT GTT GGA GAC C	60
P4ha2 V2	CGA TGA GCA AGA TGC TTT CA	CAG CTT CGA CAT CGC TCA T	48
Ndrg1	GTG CAG GGC ATG GGA TAC	TCC AGG GAT GTG ACA CTG G	29
Adm	TTC GCA GTT CCG AAA GAA GT	GGT AGC TGC TGG ATG CTT GT	103
Rplp0	ACT GGT CTA GGA CCC GAG AAG	CTC CCA CCT TGT CTC CAG TC	9

Table S2, related to Figure 6. Roche LightCycler480 Program

Target temperature (°C)	Acquisition mode	Hold	Ramp rate (°C/s)	Second Target (per °C)	Step size (°C)
**Pre-Incubation**					
94	None	7 min	4.8	0	0
**Amplification**					
94	None	15 sec	4.8	0	0
60	None	30 sec	2.5	50	0.5
72	Single	15 sec	1.5	0	0
**Cooling**					
40	None	30 sec	2.5	0	0

SUPPLEMENTAL EXPERIMENTAL PROCEDURES

Animal experiments
Administration of the hypoxia markers EF5 (60mg/kg; provided by Dr. Cameron Koch, University of Pennsylvania, PA) or pimonidazole (60 mg/kg; Hypoxyprobe, HP2-1000Kit) was by intraperitoneal injection in PBS, either 26 or 2 hours prior to tissue harvest. TH-302 (50 mg/kg; Selleckchem, S2757) was administered QDx5 by intraperitoneal injection in saline (0.9% NaCl). AZD2014 (2.5 mg/kg; Selleckchem, S2783) was administered daily by oral gavage in 30% w/v PEG400/0.5% Tween80/5% Propylene glycol, after first coating the gavage needles in sucrose solution (24%; Sigma-Aldrich, S9378) (Hoggatt et al., 2010). AZD2014 and TH-302 treatments began when tumors reached a volume of 20 mm^3 and ended at a final volume of 350 mm^3 ($\frac{length \times width^2}{2}$). Growth rate was calculated for each mouse using the linear regression fit in GraphPad Prism (GraphPad Software, Inc., CA).

Cell culture and reagents
The $KP^{fl}C$ and KPC primary PDAC cell lines (Morton et al., 2010) and telomerase immortalized fibroblasts (TIFs, (Munro et al., 2001)) were maintained in Dulbecco's modified eagle medium (DMEM; Gibco) supplemented with 10% FBS, 10 mM HEPES (Sigma-Aldrich, H0887) and penicillin/streptomycin at 100 U/ml. For stable expression of the Eevee-Akt-mT2 intramolecular FRET biosensor, KPC cells were co-transfected with pPB.DEST-Eevee-Akt-mT2 and pCMV-hyPBase (obtained from the Wellcome Trust Sanger Institute, (Yusa et al., 2011)), using Lipofectamine3000, as per the manufacturer's protocol (ThermoFisher Scientific). FACS isolated populations (FACSARIA III, BD Biosciences) with stable expression of Eevee-Akt-mT2 were isolated within a narrow fluorescence range. EGF (R&D Systems, 236-EG) stock solution was made up in water prior to use. NVP-BEZ235 (Symansis, SY-BEZ235), AZD2014 (Selleckchem, S2783), Rapamycin (LC Laboratories, R-5000), TH-302 (Selleckchem, S2757), Doxorubicin (Selleckchem, S1208) and Laurdan (Molecular Probes, D250) were made up as stock solutions in DMSO, prior to use. A H35 Hypoxystation (Don Whitley Scientific) was used for experiments conducted at 0.1% and 1% oxygen. Experiments at 5% oxygen were conducted in a HeraCell 150i CO_2/O_2 incubator. For *in vitro* pimonidazole treatments, cells or organotypic matrices were treated with pimonidazole (236 μM) 2 hours prior to harvest or fixation.

Cloning
The intramolecular FRET biosensor Eevee-Akt-mT2 (Conway et al., 2017; Goedhart et al., 2012; Komatsu et al., 2011) was subcloned into a custom Gateway-compatible Piggybac transposon-based destination vector, pPB.DEST (Vennin et al., 2017), by performing an LR reaction with pENTR221-Eevee-Akt-mT2 (Conway et al., 2017) (LR clonase II, ThermoFisher Scientific), which was then verified by an analytical digest and sequencing.

FACS
Parallel T25 flasks were seeded overnight with $1x10^5$ $KP^{fl}C$ or KPC cells, which were then grown for 48 hours in normoxic or hypoxic (0.1%, 1% and 5% oxygen) conditions. Cells were then fixed in 70% ethanol for at least 24 hours at -20^oC, prior to staining with propidium iodide (Sigma-Aldrich, P4170, 1 μg/ml) with RNaseA (Sigma-Aldrich, R6513, 500 μg/ml), and analysis on a FACS Canto II (BD Bioscience). For HistoneH3(Ser10) (Cell Signaling, 9701, 1:50) staining, cells were fixed as above, then permeabilized in PBS/0.25% Triton X-100, prior to primary antibody incubation in PBS/1% BSA at 4^oC overnight. Samples were then stained with DyLight 649-conjugated anti-rabbit secondary antibody (Jackson ImmunoResearch, 211-492-171, 1:100), propidium iodide with RNaseA, for analysis on a FACS Canto II (BD Bioscience). Annexin V (FITC)/propidium iodide (PI) staining kit (Biovision, K101) was performed as per manufactures instructions. Quantification was performed in FlowJo (Tree Star) and cell cycle phase determined in ModFit (Verity Software House).

Immunofluorescence (IF)
O.C.T. (Tissue-Plus O.C.T. compound, Scigen, 4583) embedded tumours were cryo-sectioned at 4 μm and fixed for 20 minutes with ice-cold 4% PFA. Sections then underwent a 10 minute Protein Block (Dako, X0909), prior to overnight incubation with FITC-conjugated pimonidazole (Hypoxyprobe, 1:25) and Cy5-conjugated EF5 (ELK3-51, 75 μg/ml; purchased from Dr. Cameron Koch, University of Pennsylvania, Philadelphia, PA) primary antibodies. For IF with Akt(Ser473) (Cell Signaling, 4060, 1:100), HIF1α (Novusbio, NB100-449, 1:300) or Ki67(SP6) (ThermoFisher Scientific, RM-9106-S1, 1:200), blocking was performed for 1 hour in blocking buffer (10% normal donkey serum (Jackson

ImmunoResearch, 017-000-121), 2.5% BSA (Sigma-Aldrich, A7906), 0.02% glycine and 0.3% Triton X-100), then incubated with Alexa Fluor 647-conjugated anti-rabbit secondary antibody (Jackson ImmunoResearch, 711-606-152, 1:500) for 1 hour and DAPI for 10 minutes. IF samples were imaged on a Leica DMI 6000 SP8 microscope with a 25X water objective using 488 nm and 633 nm continuous wave lasers for FITC and Cy5/Alexa Fluor 647 excitation respectively. DAPI was excited at 750 nm with a Ti:Sapphire femtosecond laser (Coherent, Chameleon Ultra II). Signal overlap between the FITC and Cy5 channels was quantified using the colour threshold function in ImageJ (NIH). Quantification of the Akt(Ser473) grey value/cell, as well as Ki67 positive and negative nuclei in pimonidazole positive and negative regions was performed in QuPath (Bankhead et al., 2017). For quantification of HIF1α staining, a nuclear mask was first generated in ImageJ, prior to quantification of the grey value/nucleus for each image, and averaging this over 5 regions of 232x232 μm. For quantification of pimonidazole staining, the mean grey value was calculated for 5 regions of 232x232 μm, and divided by the number of cells in the image using ImageJ.

Immunohistochemistry (IHC)

Formalin fixed paraffin embedded organotypic matrices and tissues were used to cut 4 μm sections prior to either haematoxylin and eosin (H&E) staining on a Leica Autostainer or IHC staining for Ki67(SP6) (ThermoFisher Scientific, RM-9106-S1, 1:500), pan-cytokeratin (Leica-Novocastra, NCL-C11, 1:50), HIF1α (Novusbio, NB100-449, 1:300), γH2AX(Ser139) (Cell Signaling, 9718, 1:500), GLUT1 (Abcam, ab652, 1:1,000), lactate dehydrogenase (Abcam, 1:2,000, ab52488), NDRG1(Thr346) (Cell Signaling, 5482, 1:1,000), HistoneH3(Ser10) (Cell Signaling, 9701, 1:100), CD31 (Dianova, DIA-310, 1:100), CAIX (Novusbio, NB100-417, 1:500). Staining for pimonidazole adducts was performed using a FITC-conjugated pimonidazole primary antibody (Hypoxyprobe, 1:1,000) and an anti-FITC secondary antibody (Hypoxyprobe, 1:100). An Aperio S2 ScanScope (Leica Biosystems) or a DM4000 (Leica Biosystems) was used for imaging H&E or IHC staining. For quantification of Ki67, γH2AX(Ser139), HistoneH3(Ser10) and HIF1α staining, positive cells were counted and divided by the total cell number in 500x500 μm images (5 representative regions/tumour section). Additional phenotypic scoring of HistoneH3(Ser10) positive cells was performed to classify cells into late G_2, prophase, metaphase and anaphase. Quantification of CAIX stain coverage was performed in ImageJ (NIH) by first using colour deconvolution to isolate the DAB stained area, then measuring the stain coverage in 500x500 μm images (10 representative regions/tumour section). To measure the necrotic fraction of tumours, whole tumour images were taken and the necrotic region traced in ImageJ (NIH). To measure the hypoxic fraction of tumours, whole tumour images were taken and pimonidazole isolated in ImageJ (NIH) by first using colour deconvolution to isolate the DAB stained area, then measuring the staining coverage. Cells with fragmented nuclei were scored on H&E stained sections, as described previously (Vennin et al., 2017), selecting 6 regions/tumour of 848x478 μm for analysis.

Intravital FLIM/PLIM imaging

Xenografts of KPC cells expressing the Eevee-Akt-mT2 FRET biosensor imaged at a final volume of 350 mm^3, as per the survival studies. At this point, mice were anesthetized with Xylazine (10 mg/kg) and Zoletil (50 mg/kg) and kept on a heated stage at 37°C. Anesthesia was maintained with isoflurane (3L, O2 1L, vacuum 1L/min). Tumours were surgically exposed, as described previously (Conway et al., 2017; Nobis et al., 2013), prior to application of MitoImage NanO2 (10 μg; Ibidi, 74151). After 30 minutes, dual FLIM/PLIM imaging was performed on a Leica DMI 6000 SP8 microscope with a 0.95 NA 25X water objective. FLIM-FRET imaging was performed using excitation at 840 nm with a Ti:Sapphire femtosecond laser (Coherent, Chameleon Ultra II). Emitted fluorescence was recorded using a RLD HyD detector with a 483/32 nm filter. Photon arrival times were recorded using a TCSPC system (Picoharp 300, Picoquant). 512 x 512 pixel images were acquired with a line rate of 700 Hz and integrated over 242 frames. PLIM imaging of MitoImage NanoO2 was performed with excitation at 760 nm. 128 x 128 pixel images were recorded at a line rate of 10 Hz, giving a pixel dwell time of 225 μs. An EOM was used to modulate the excitation synchronously with the pixel clock with a 1:16 duty cycle, producing 14 μs pulses at the start of each pixel. The phosphorescence decay was recorded using an RLD HyD detector with a 650/50 nm. Photon arrival times relative to the pixel start time were recorded using a time to digital converter (TimeTagger4-2G, Cronologic). Photon arrival times were recorded with a resolution of 1024 ns. Analysis of both FLIM and PLIM decays was performed in FLIMfit (Conway et al., 2017; Warren et al., 2013) for >23 cells/mouse using a maximum likelihood estimator. For FLIM data, background autofluorescence was taken into account using a time varying background (see "Correction for autofluorescence in *in vitro* and *in vivo* FLIM data" section). Lifetime maps were generated from the raw data after smoothing with a 3x3 pixel kernel and applying a standard rainbow colour look-up table (LUT). To exclude areas where no lifetime measurement above

the background noise could be achieved, the intensity threshold was set to the average background pixel value for each recording and these areas are shown in black.

Live cell imaging

KPC PDAC cells expressing either Eevee-Akt-mT2 were seeded at $2x10^5$ cells/FluoroDish (World Precision Instruments, FD35-100) 2 days prior to imaging. Imaging was performed on a Leica DMI 6000 SP8 microscope with a 0.95 NA 25X water objective, within a 37°C incubator, supplemented with 5% CO_2. FLIM-FRET and PLIM imaging were performed as per the "Intravital FLIM/PLIM imaging" section above. For nanoparticle imaging, FluoroDishes were incubated overnight with MitoImage NanO2 (4 µg; Ibidi, 74151) or MM2 (4 µg; Ibidi, 74161) and media changed just prior to imaging. To reduce oxygen levels in culture, 25 mm glass coverslips (#1.5, Warner Instruments, 64-0715) were gently placed on top of the cell monolayer for 1 and 2 hours, as previously described (Pitts and Toombs, 2004; Takahashi et al., 2012). Ratiometric imaging of MitoImage MM2 was performed at 760 nm, on two RLD HyD detectors (440/20 nm (PFO) and 650/50 nm (PtTFPP)), at a scan speed of 10 Hz. To assess the ratio of PtTFPP to PFO, the channels were overlaid and the intensity ratio calculated in ImageJ (NIH). For FLIM-FRET at 0.1%, 1% and 5% oxygen, KPC cells were fixed in 4% PFA prior to imaging, along with their respective normoxic (21%) control.

Correction for autofluorescence in *in vitro* and *in vivo* FLIM data

The small molecule inhibitor AZD2014 was found to be weakly fluorescent in the 483/32 nm emission range used for FLIM-FRET analysis of the Eevee-Akt-mT2 FRET biosensor. By analyzing the fluorescence lifetime of KPC cells treated with high (2 µM) concentrations of AZD2014 we determined that the lifetime of AZD2014 was 5.429 ± 0.027 ns (Figure S5G). If not taken into account, this drug autofluorescence would introduce an intensity-dependent artifact into the FLIM analysis, biasing the lifetime towards the drug's fluorescence lifetime more strongly in pixels where the biosensor fluorescence is less intense (see bias in intensity-lifetime correlation plot in Figure S5H). To correct for this autofluorescence, we included a time-varying background (TVB) in our FLIM analysis model, as previously described (Conway et al., 2017; Warren et al., 2013). To measure the TVB, we used untransfected cells (*in vitro*) or stromal cells (*in vivo*) in the same treatment well (*in vitro*) or tumour (*in vivo*). By using a TVB from a neighboring region the appropriate drug concentration is taken into account (producing the expected symmetrical distribution in the intensity-lifetime correlation plot, see Figure S5H). To validate our correction approach, we plotted the intensity of each cell against its' lifetime and fitted a linear model (Figure S5I). If there were an intensity-dependent lifetime artifact due to the drug autofluorescence we would expect a negative slope, as cells with a lower biosensor fluorescence intensity would be biased towards the longer drug lifetime. In the absence of an artifact, we would expect the slopes to be zero on average, as observed (Figure S5I). Using *in vitro* data treated with DMSO, 500 nM and 1µM AZD2014 we found that the slopes were not significantly different from zero, indicating our correction was effective (Figure S5I).

Organotypic assay

Organotypic matrices were generated as described previously (Conway et al., 2014; Morton et al., 2010; Timpson et al., 2011; Vennin et al., 2017). Briefly, ~$1x10^5$ TIFs/matrix were embedded in acid-extracted rat tail tendon collagen (~2 mg/ml), which was prepared by extracting fresh frozen rat tail tendons in 0.5 M acetic acid. After neutralizing with NaOH and in the presence of 1X MEM (Gibco, 11430-030), matrices were allowed to set at 37°C. Detached polymerized matrices were then allowed to contract for 12 days, prior to seeding with $1x10^5$ KPC cells/matrix. After 4 days, seeded matrices were moved to an air-liquid interface to invade for 12 or 14 days, as detailed below.
For 12 day invasions, vehicle (DMSO) or the PI3K-pathway inhibitors NVP-BEZ235 (100 nM), AZD2014 (500 nM) or rapamycin (100 nM), were added for the final 6 days of the invasion. For 14 day invasions, cells were allowed to invade for 11 days, prior to 3 days with either TH-302 (1 µM), AZD2014 (500 nM) or both in combination. Pimonidazole (60 mg/L, Hypoxyprobe) was added to the media for 2 hours prior to fixation in 10% neutral buffered formalin and paraffin block embedding and sectioning. Quantification of the invasive index, as well as Ki67, NDRG1(Thr346) and γH2AX(Ser139) positive cells, was performed on pan-cytokeratin (excludes fibroblasts) or Ki67, NDRG1(Thr346) or γH2AX(Ser139) stained sections respectively. From each condition and replicate, 10 representative 500x500 µm regions were selected. To calculate the invasive index, invaded cells were counted and divided by the sum of the invaded cells and cells on the surface of the matrix. Similarly, the number of positive cells was calculated as the ratio of Ki67, NDRG1(Thr346) or γH2AX(Ser139) positive cells, divided by the total cell number in each area. These indices were then normalized to the DMSO controls for each respective treatment. For assessment of the diffusion of

laurdan and doxorubicin into the organotypic matrices, each compound was added to the liquid phase for 2 hours, prior to imaging on a Leica DMI 6000 SP8 microscope with a 0.95 NA 25X water objective, within a 37°C incubator, supplemented with 5% CO_2. Laurdan and doxorubicin were excited at 780 nm and 740 nm respectively, using a Ti:Sapphire femtosecond laser (Coherent, Chameleon Ultra II). Detection of Laurdan or Doxorubicin fluorescence was performed on a RLD HyD detector using a 483/32 nm or 585/40 nm filter respectively. Second harmonic generation (SHG) imaging of organotypic matrices was performed at 890 nm, detecting SHG intensity with an RLD HyD detector with a 440/20 nm filter.

Growth assays

Parallel 24-well plates were seeded for measurements in both normoxic and hypoxic (0.1% oxygen) conditions, for days 0-3 of the growth curves. At each day of imaging, one plate was fixed in 4% PFA and stained with Hoechst 33342 (Molecular Probes, H1399, 1 μg/ml). Six wells per plate were imaged for each condition on a Leica DMI 6000 SP8 microscope with a 10X dry objective. At least 5 images/well were quantified in QuPath using positive cell detection (Bankhead et al., 2017).

Quantification of vessels in PDAC xenografts

Amira 6 software (FEI, Thermo Scientific) was used for morphometric quantification of CD31 stained xenografts after light inversion in Photoshop (Adobe). Similar to previous work (Vennin et al., 2017), images were segmented using a combination of automatic and manual selections, available in Amira 6. Segmented images could then be assessed for coverage of CD31 stained microvessels. The dimensions per pixel were calculated by dividing the micron ratio by the pixel ratio for each image, to give coverage in μm^2 per image.

RNA isolation, reverse transcription and quantitative RT-PCR (qRT-PCR) experiments and analysis

KPC cells were seeded at $2x10^5$ cells/6-well well and grown for 48 hours prior to incubation for 2 hours in normoxia, hypoxia (5%, 1% and 0.1% oxygen), or under a coverslip, and collection in QIAzol lysis reagent (Qiagen). RNA samples were isolated using the miRNeasy kit (Qiagen) and reverse transcribed with the Transcriptor First Strand cDNA Synthesis Kit (Roche Diagnostics). cDNA was synthesized from 1 μg of total RNA and diluted 1:10 before any further analysis. qRT-PCR experiments were performed using the Roche Universal Probe Library System on a Roche LightCycler480® (Roche LifeScience). Probes and programs used for qRT-PCR analysis are listed in Tables S1 and S2. Relative mRNA expression levels were normalized to *Rplp0* and quantification was performed using the comparative C_T method (Schmittgen and Livak, 2008).

Western Blotting

Cell lysates were prepared in RIPA lysis buffer (50 mM HEPES, 1% Triton X-100, 0.5% sodium deoxycholate, 0.1% SDS, 0.5 mM EDTA, 50 mM NaF, 10 mM Na_3VO_4, and protease inhibitor cocktail (cOmplete™ Mini, EDTA-free, Roche)) and volumes adjusted according to protein concentration measurements (Bio-Rad Protein Assay Dye Reagent Concentrate, Bio-Rad, 5000006). Separation was performed by gel electrophoresis (NuPage™ 4-12% Bis-Tris Protein Gels, ThermoFisher Scientific, NP0336), prior to transfer onto a PVDF membrane (Immobilon-P, Millipore) and blocking in 5% skim milk. Primary antibodies in TBS/BSA for HIF1α (Novusbio, NB100-449, 1:1,000), Akt(Ser473) (Cell Signaling, 9271, 1:1,000), Akt(Thr308) (Cell Signaling, 9275, 1:1,000), Akt (Cell Signaling, 9272, 1:1,000), p70-S6K(Thr389) (Cell Signaling, 9205, 1:1,000), p70-S6K (Cell Signaling, 9202, 1:1,000), FITC-conjugated pimonidazole primary antibody (Hypoxyprobe, 1:10,000), GLUT1 (Abcam, ab652, 1:1,000), lactate dehydrogenase (Abcam, ab52488, 1:2,000), NDRG1(Thr346) (Cell Signaling, 5482, 1:1,000), p53 (Calbiochem, OP03L, 1:1,000), NDRG1 (Cell Signaling, 5196, 1:1,000) or β-actin (Sigma Aldrich, A5441, 1:10,000) were incubated overnight at 4°C. Signal was detected by first incubating TBST washed membranes with HRP-linked secondary antibodies (GE Healthcare, 1:5,000 diluted in 1% skim milk) for at least 1 hour at RT, then applying ECL reagent (Western Lightning Plus-ECL, PerkinElmer, NEL104001EA) and imaging on a Fusion FX (Vilber). For detection of pimonidazole primary antibody, anti-FITC HRP secondary antibody (Hypoxyprobe, 1:5,000) was used, following the procedure above. Densitometry was performed in ImageJ (NIH) normalizing signal to either β-actin or the respective total protein.

SUPPLEMENTAL REFERENCES

Bankhead, P., Loughrey, M.B., Fernandez, J.A., Dombrowski, Y., McArt, D.G., Dunne, P.D., McQuaid, S., Gray, R.T., Murray, L.J., Coleman, H.G., et al. (2017). QuPath: Open source software for digital pathology image analysis. Scientific reports *7*, 16878.

Goedhart, J., von Stetten, D., Noirclerc-Savoye, M., Lelimousin, M., Joosen, L., Hink, M.A., van Weeren, L., Gadella, T.W., Jr., and Royant, A. (2012). Structure-guided evolution of cyan fluorescent proteins towards a quantum yield of 93%. Nature communications *3*, 751.

Hoggatt, A.F., Hoggatt, J., Honerlaw, M., and Pelus, L.M. (2010). A spoonful of sugar helps the medicine go down: a novel technique to improve oral gavage in mice. J Am Assoc Lab Anim Sci *49*, 329-334.

Munro, J., Steeghs, K., Morrison, V., Ireland, H., and Parkinson, E.K. (2001). Human fibroblast replicative senescence can occur in the absence of extensive cell division and short telomeres. Oncogene *20*, 3541-3552.

Nobis, M., McGhee, E.J., Morton, J.P., Schwarz, J.P., Karim, S.A., Quinn, J., Edward, M., Campbell, A.D., McGarry, L.C., Evans, T.R., et al. (2013). Intravital FLIM-FRET imaging reveals dasatinib-induced spatial control of src in pancreatic cancer. Cancer research *73*, 4674-4686.

Pitts, K.R., and Toombs, C.F. (2004). Coverslip hypoxia: a novel method for studying cardiac myocyte hypoxia and ischemia in vitro. American journal of physiology Heart and circulatory physiology *287*, H1801-1812.

Schmittgen, T.D., and Livak, K.J. (2008). Analyzing real-time PCR data by the comparative CT method. Nat Protocols *3*, 1101-1108.

Takahashi, S., Piao, W., Matsumura, Y., Komatsu, T., Ueno, T., Terai, T., Kamachi, T., Kohno, M., Nagano, T., and Hanaoka, K. (2012). Reversible off-on fluorescence probe for hypoxia and imaging of hypoxia-normoxia cycles in live cells. Journal of the American Chemical Society *134*, 19588-19591.

Timpson, P., McGhee, E.J., Erami, Z., Nobis, M., Quinn, J.A., Edward, M., and Anderson, K.I. (2011). Organotypic collagen I assay: a malleable platform to assess cell behaviour in a 3-dimensional context. J Vis Exp, e3089.

Yusa, K., Zhou, L., Li, M.A., Bradley, A., and Craig, N.L. (2011). A hyperactive piggyBac transposase for mammalian applications. Proc Natl Acad Sci U S A *108*, 1531-1536.
